# Identification of a Quaternary rock avalanche deposit (Central Apennines, Italy): Significance for recognition of fossil catastrophic mass‐wasting

**DOI:** 10.1111/sed.12984

**Published:** 2022-03-29

**Authors:** Diethard Sanders, Teresa Dendorfer, R. Lawrence Edwards, Gina E. Moseley, Hugo Ortner, Simon Steidle

**Affiliations:** ^1^ Department of Geology University of Innsbruck Innrain 52f Innsbruck A‐6020 Austria; ^2^ School of Earth and Environmental Sciences University of Minnesota 116 Church Street Minneapolis MN 55455‐0149 USA; ^3^ Present address: Staatliches Bauamt Ingolstadt Elbrachtstraße 20 Ingolstadt D‐85049 Germany

**Keywords:** Apennines, Campo Imperatore, intramontane basin, rock avalanche, U/Th

## Abstract

Whereas deposits of extremely‐rapid, ‘catastrophic’ mass wastings >10^5^ m^3^ in volume (for example, the Marocche di Dro rock avalanche in the Southern Alps and the Flims rockslide in the Western Alps) are easily recognized by their sheer mass and blocky surface, the identification of *fossil* catastrophic mass wastings partly removed by erosion must be based on deposit characteristics. Herein, a ‘fossil’ (pre‐last glacial) rock avalanche, previously interpreted as either a till or debris flow, is described. The deposit, informally called ‘Rubble Breccia’, is located in the intramontane Campo Imperatore halfgraben that is bounded by a master fault with up to *ca* 900 m topographic throw. Based on documentation from field to thin section, and by comparative analysis with post‐glacial rock avalanches, tills and debris flows, the Rubble Breccia is reinterpreted as a rock avalanche. The Rubble Breccia consists of an extremely‐poorly sorted, disordered mixture of angular clasts from sand to block size. Many clasts show fitted subclast boundaries in crackle, jigsaw and mosaic fabrics, as diagnostic of catastrophic mass wasting deposits. Intercalated layers of angular to well‐rounded clasts of coarse sand to fine pebble size, and deformed into open to recumbent folds, may represent shear belts folded during terminal avalanche propagation. The clast spectrum of the Rubble Breccia – mainly shallow‐water bioclastic limestones, *Saccocoma* wackestones and other deep‐water limestones and dolostones – is derived from the front range along the northern margin of the basin. Calcite cement found within the Rubble Breccia was dated with the U/Th disequilibrium method to 124.25 ± 2.76 ka bp, providing an *ante‐quam* age constraint to the rock avalanche event. Because catastrophic mass wasting is a common erosional process, fossil deposits thereof should be more widespread than have been identified to date, although this may be a consequence of misidentification. The criteria outlined here provide a template to identify fossil catastrophic mass wasting deposits of any age.

## INTRODUCTION

Catastrophic mass‐wasting (CMW; rock avalanches, rockslides) is a characteristic process of erosion operating on mountain ranges (Densmore *et al*., 1997; Malamud *et al*., 2004; Ouimet *et al*., 2007; Korup *et al*., 2010; Ekström & Stark, 2013), hence examples are abundant worldwide. Catastrophic mass‐wasting events are defined as extremely rapidly (>5 m s^−1^) (Hungr *et al*., 2001) propagating mass‐wastings of rock with a volume of at least *ca* 10^5^ m^3^ that undergo dynamic fracturation of the rock during transport (Davies & McSaveney, 2002; Evans *et al*., 2006; Hungr *et al*., 2012; Schilirò *et al*., 2019; Davies *et al*., 2020). It is well‐established that the incidence of CMWs is favoured by; (i) high topographic relief, combined with (ii) structural predisposition of the rock, such as intersecting faults and joints, or dip slopes parallel to – each or either of – faults/joints, stratification or schistosity, combined with (iii) coseismic ground acceleration and/or heavy rains, removal of slope support (for example, by stream incision), karstification, or halokinetic deformation (Longwell, 1951; Whitehouse & Griffiths, 1983; Keefer, 1984; Schuster *et al*., 2002; Sartori *et al*., 2003; Jibson *et al*., 2006; Strom & Korup, 2006; Lenhardt, 2007; Prager *et al*., 2009; Crosta *et al*., 2012; Yin *et al*., 2012; Ekström & Stark, 2013; Lin *et al*., 2015; Luo *et al*., 2019; Rossato *et al*., 2020; Sanders *et al*., 2021; Wang *et al*., 2021, and many others). This combination is realized in a wide variety of tectonic settings, such as convergent margins (Schuster *et al*., 2002; Antinao & Gosse, 2009; Mather *et al*., 2014; Crosta *et al*., 2017; Arai & Chigira, 2018; Rouhi *et al*., 2019), oblique‐convergent margins and/or large strike‐slip faults (Hung *et al*., 2002; Dufresne *et al*., 2009; Allen *et al*., 2010; Halliday & Camara, 2012; Barth, 2014), in intraplate orogens and mature orogens undergoing strike‐slip faulting, such as the Alps (Strom & Korup, 2006; Prager *et al*., 2008; Brideau *et al*., 2009; Ostermann & Sanders, 2012, 2016; Oswald *et al*., 2021; see Hewitt *et al*., 2008, for compilation), and in orogens undergoing extensional faulting behind their leading compressional front, such as the Apennines (Galadini, 2006; Bianchi Fasani *et al*., 2014; Antonielli *et al*., 2020).

Because of the sheer volumes of rock (up to tens of Gm^3^) involved in CMWs (e.g. Crandell *et al*., 1984; Rouhi *et al*., 2019), vestiges of ‘fossil' (= pre‐last glacial) CMWs may be expected in degrading mountain ranges and in intramontane basins. Fossil CMWs thus are purportedly widespread yet, except for a few case studies (Longwell, 1951; Mudge, 1965; Schultz, 1986; Topping, 1993; Yarnold, 1993; Friedmann, 1997; Gruber *et al*., 2009; Antonielli *et al*., 2020), appear to be largely absent in the pre‐last glacial record, possibly due to real scarcity and/or due to nonrecognition of this type of deposit in the older geological record. Fossil ‘landslides' and CMWs are no longer obvious by their typically blocky surface and bulky shape of the entire deposit, but have been modified by different geomorphological processes (see, e.g., Topping, 1993; Yarnold, 1993; Mather *et al*., 2003; Evans *et al*., 2006; Hewitt *et al*., 2008; Gruber *et al*., 2009; Antonielli *et al*., 2020).

The Apennines mountain range in Italy is characterized by widespread normal faulting and development of intramontane basins that persisted for millions of years (e.g. Cavinato *et al*., 2002; Gori *et al*., 2017). For the Apennines, a clear‐cut association of CMWs with: (i) stratal dip slopes along the leading edge of folds and thrusts; and (ii) with large normal faults that subsequently formed along the trailing side of the thrusts is well‐established (Galadini, 2006; Bianchi Fasani *et al*., 2014; Antonielli *et al*., 2020). Because the normal faults mediate the formation of intramontane basins, the CMWs associated with downfaulting should be of comparatively high geological preservation potential. Geologically ‘young' (post‐last glacial) CMW deposits preserved at the surface display several characteristics that render them readily identifiable, such as: (i) an unusually thick sheet with a relief up to a few tens of metres and/or a very thick bulky deposit that blocks – or formerly blocked – a valley; combined with (ii) a surface littered with boulders to blocks of lithologies derived from a detachment scar that; (iii) typically is obvious as a more or less niche‐shaped morphological recess. Fossil CMW deposits that are only partly preserved and/or partly exposed in laterally limited outcrops, however, lack the distinct surface morphology displayed by young CMWs; in addition, depending on the age of a fossil CMW, the detachment area may also have undergone modification by erosion after the mass‐wasting event. In consequence, it seems that fossil CMWs were often ascribed to other types of fragmentites or poorly‐sorted coarse‐grained deposits, such as fault cataclasites, glacial deposits or debris flows. Incompletely preserved and/or incompletely exposed fossil CMWs thus must be identified on the basis of criteria related to sediment fabric.

**Fig. 1:**
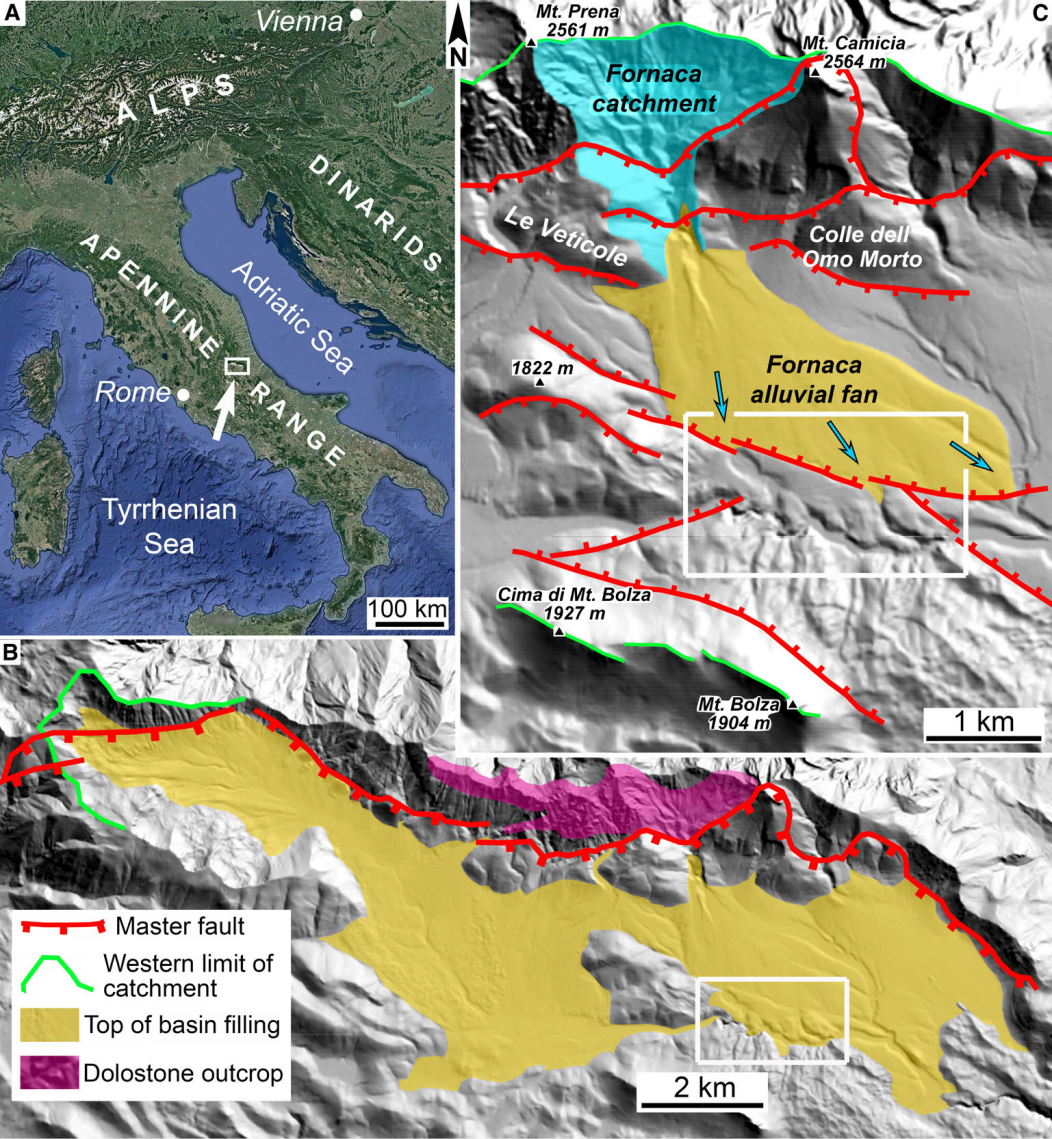
(A) Position of study area (white rectangle labelled by arrow) in Italy. (B) LIDAR (light detection and ranging) topography of Campo Imperatore basin. The main outcrop of dolostones (cf. Table 1) is schematic after Vezzani & Ghisetti (1998). White rectangle marks the area shown in Fig. 3. (C) Detail of (B), showing master fault and synthetic and antithetic faults across the basin width. The Fornaca catchment supplies the large Fornaca alluvial fan that is relevant herein. White rectangle marks the area shown in Fig. 3.

Hereunder, based on a set of criteria to distinguish different types of coarse‐grained fragmental deposits in laterally limited exposures, a deposit previously interpreted as a glacial diamicton or a debris‐flow deposit is reinterpreted as a fossil rock avalanche. The studied deposit is located in the Campo Imperatore intramontane basin of the central Apennines (Fig. 1). Based on; (i) a comparison with ‘young’ (post‐last glacial) rock avalanches and with other deposits that contain fracturated clasts, and with (ii) documented or deduced processes operating within CMWs, criteria on a scale ranging from outcrops (typically a few tens of metres to hundreds of metres) down to thin sections of how to identify a fossil rock avalanche deposit (RAD) that is no longer evident by surface morphology are developed. A U/Th disequilibrium age of cement within the RAD highlights the potential of this method to derive *ante‐quam* age constraints on geological processes as well as of the CMW deposits themselves (cf. Sanders *et al*., 2010; Ostermann *et al*., 2017). In the Apennines, as well as in other mountain ranges, deposits of fossil CMWs should be more common than appreciated as yet, but perhaps were overlooked or mistaken for other rock types. Systematic reinvestigation of coarse‐grained deposits that might be candidates for fossil CMW deposits based on the criteria outlined herein should help to detect these.

## SETTING

**Fig. 2:**
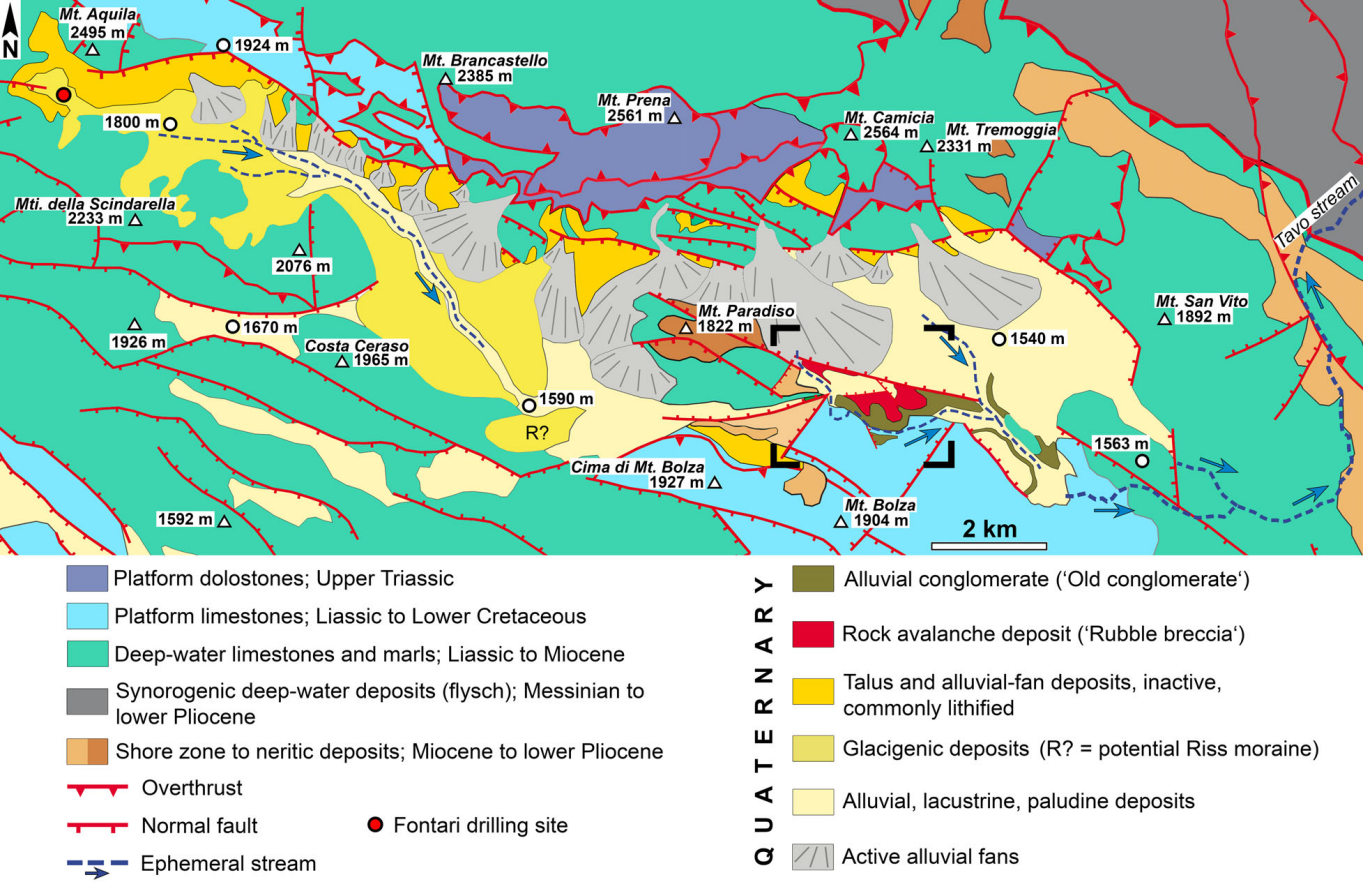
Geological map of the Campo Imperatore basin (simplified from Ghisetti & Vezzani, 1986). The halfgraben basin *ca* 16 km in length is filled with Quaternary deposits, and is bounded along its northern front range by a system of major normal faults (Campo Imperatore fault system). The studied rock‐avalanche deposit is exposed in the eastern part of the basin. The black rectangle corners denote the area of the map shown in Fig. 3.

The central Apennines are a sector of the NNW–SSE trending mountain range that backbones the Italian peninsula, and consist mainly of Meso‐Cainozoic carbonate rocks from shallow‐water platform to pelagic basinal settings (Figs 1A and 2) (Cardello & Doglioni, 2015). In the central Apennines, piggyback basins record eastward‐directed, late Burdigalian to late Pliocene shortening (Cosentino *et al*., 2017). While shortening took place in the external part of the orogen, in more internal positions, formation of extensional basins started (Cipollari *et al*., 1999). Most of the extensional basins of the central Apennines originated during the late Piacenzian to early Gelasian (Cosentino *et al*., 2017, fig. 13). The hiatuses between contractional/extensional deformation seem to be neither of similar duration nor of systematic distribution (with respect to duration) among different basins (Cosentino *et al*., 2017, p. 1330). For the central Apennines, Cosentino *et al*. (2017) distinguish a single, late Pliocene to earliest Pleistocene rifting phase. Unfortunately, the onset of filling of the Campo Imperatore basin is not dated. Most of the major, low‐positioned intramontane basins filled with Pliocene to Holocene successions have acted as sediment traps until the present (Cavinato *et al*., 2002; Galadini *et al*., 2003; Gori *et al*., 2014), or have undergone moderate uplift and erosion (Gori *et al*., 2017). Basins positioned at higher altitudes, in turn, typically are underfilled, and the amplitude of topographic relief created by fault horsts positively correlates with fault throw (Pizzi, 2003). The flanks of the fault‐bounded basins are onlapped by scree slopes and/or alluvial fans (Giraudi, 1995; D'Agostino *et al*., 1998; D'Alessandro *et al*., 2003; Galadini *et al*., 2003; Sanders *et al*., 2018a).

In the area of the Campo Imperatore basin, Meso‐Cainozoic carbonate platform to basinal successions became stacked during the late Messinian into thrust imbricates; thrusting was followed by Pliocene to present uplift and extension (Figs 1 and 2; Table 1) (Ghisetti & Vezzani, 1999; Cardello & Doglioni, 2015). Today, the entire Gran Sasso area comprises several major normal fault systems, in part linked by transfer faults, that together comprise the largest normal fault system of the Apennines (Galli *et al*., 2022). Of these, in the present context, only the Campo Imperatore fault system is of interest that delimits an intramontane basin individuated during the Early Pleistocene (Figs 1 and 2). Aside from a few antithetic faults of comparatively small throw, the basin essentially is a halfgraben with a range front in the North (Figs 1B and 2) (D'Agostino *et al*., 1998; Ghisetti & Vezzani, 1986; Calamita *et al*., 2010). Whereas the eastern and central segment of the Campo Imperatore fault system are dormant, the segment west of Monte Brancastello (see Fig. 2) shows clear‐cut evidence for recent activity (Demurtas *et al*., 2016; Ortner *et al*., 2018; Galli *et al*., 2022). To date, only a single borehole exists in the westernmost part of the Campo Imperatore basin (Fig. 2). Before reaching the rock substrate, this drilling had penetrated 176 m of a Quaternary succession characterized as ‘mainly morainic’, and consisting of clasts identical to the lithologies exposed on the basin flanks (Calembert *et al*., 1972).

**Table 1:** Stratigraphic units, lithologies and ages exposed in the catchment of the Campo Imperatore basin, arranged according to age. Lithologies briefly characterized (see Ghisetti & Vezzani, 1986) are grouped according to deposition in shallow (euphotic) waters or in deep waters of slope to basinal settings. Units exposed: (i) along the northern front range of the Campo Imperatore basin, and within a north–south sector delimited by the summits of Monte Prena and Monte Tremoggia (see Fig. 3), are shown **bold red** ; (ii) units exposed along the southern basin margin (Monte Bolza to Cima di Monte Bolza) are shown **bold blue** .

Formation	Depositional setting lithology	Age remarks
**Dolomia Principale**	**Shallow water platform** Typically medium to coarse‐crystalline dolostones	Norian to Rhaetian
**Dolomie Bituminose**	**Deep water, intraplatform basin** Thin‐bedded, typically fine to medium crystalline dolostones with levels of black organic matter; intercalated breccias and calciturbidites.	Norian to Rhaetian
**Calcari Selciferi and equivalents**	**Deep water** Cherty lime mudstones with ammonites and calciturbidites	Hettangian to Sinemurian
**Calcare Massiccio**	**Shallow water platform** Stacked peritidal carbonate cycles. Subtidal cycle part with oolites, corals, calcareous algae	Hettangian to Sinemurian Partly dolomitized in the westernmost sector of the basin (dolomitized portions not shown in Fig. 2)
Verde Ammonitico, Corniola	**Deep water** Marly, nodular lime mudstones and marls with ammonites, brachiopods, sponge spicules, radiolarians; locally calciturbidite beds	Pliensbachian to Aalenian
**Formazione della Terratta**	**Shallow water platform** Shallow‐water bioclastic packstone to rudstone, oolites, boundstone	Upper Liassic to Lower Cretaceous
**Calcareniti ad Entrochi**	**Deep water, proximal platform slope** Shallow‐water bioclastic packstones to rudstones	Dogger to Malm
Maiolica	**Deep water** Whitish, cherty lime mudstones with radiolarians, sponge spicules and calpionellids	Tithonian to Barremian
**Calciruditi a Rudiste, Marne a Fucoidi**	**Deep water** Shallow‐water bioclastic turbidites, locally interbedded with marly limestones and marls with planktonic foraminifera	Aptian to lower Cenomanian
Scaglia equivalente, Scaglia Cinerea equivalente	**Deep water** Cherty marls and limestones with planktonic foraminifera, locally with calciturbidite beds	Upper Cretaceous to Oligocene
**Calcareniti Bioclastiche**	**Neritic, shelfal** Calcarenites with larger benthic foraminifera, marls with bryozoans and bivalves	Lower to Middle Miocene
**Calcareniti a Briozoi e Litotamni**	**Shore zone to shallow neritic** (Marly) calcarenites with coralline algae, bryozoans and larger benthic foraminifera	Lower to Middle Miocene
Conglomerati di Monte Coppe	**Shore zone** Polymictic conglomerates with matrix of lithic sand	? Messinian to lower Pliocene
**Calciruditi di Rigopiano**	**Shore zone** Carbonate‐lithic conglomerates to pebbly grainstone/packstones with shallow‐water bioclastic material	Lower Pliocene

The eastern part of the basin that is relevant herein is bounded along its northern flank by a front range between approximately 600 to 700 m in topographic relief, containing the Campo Imperatore master fault (Fig. 1B). The fault footwall along the northern basin flank became differentiated by erosion into deeply incised alluvial‐fan catchments (for example, Fornaca, Vallone di Vradda) and large projecting slope facets (Le Veticole, Colle dell Omo Morto) (Fig. 1C). Along the northern basin flank, aside from Quaternary scree slopes and alluvial fan deposits, dolostones and deep‐water limestones of Norian to Paleogene age are exposed (see Fig. 2, Table 1). The southern basin flank, in turn, is characterized by the west–east trending range of Cima di Monte Bolza and Monte Bolza 300 to 350 m in topographic relief (Fig. 1C), consisting of Hettangian to Early Cretaceous platform limestones unconformably overlain by Miocene to early Pliocene shore zone to neritic deposits (Fig. 2, Table 1). Eastward of Monte Bolza, a hilly karstic terrain incised into platform limestones reaches an altitude between approximately 1550 to 1600 m a.s.l. (above sea level). During the Last Glacial Maximum (LGM = Campo Imperatore Stadial in the central Apennines; Giraudi, 2012), a glacier supplied mainly from cirques west of Mt. Aquila and north of the Mti. della Scindarella had advanced towards the east, down to approximately half the extent of the Campo Imperatore basin (Fig. 2). This glacier deposited a laterally wide array of recessional moraines along its former terminus. An older remnant of till (labelled "R?" in Fig. 2) perhaps originated during the penultimate (Riss) glaciation.

**Fig. 3:**
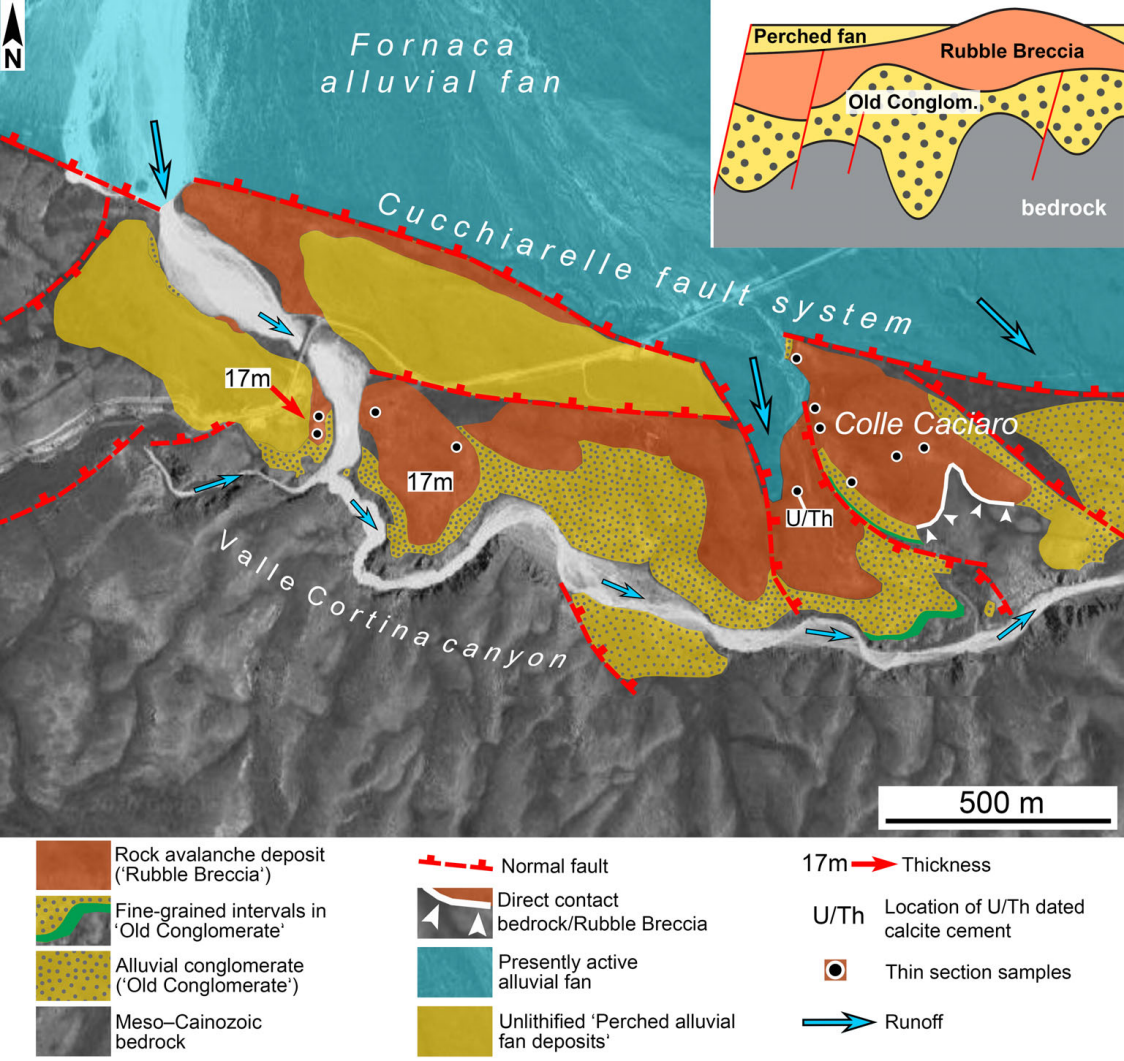
Geological map showing the depositional and structural context of the rock avalanche deposit described herein. Inset in upper right schematically shows stratigraphic relations between the Pleistocene deposits (see Table 2). At Colle Caciaro, the Old Conglomerate locally pinches out, and the Rubble Breccia directly overlies the older bedrock of Lower Jurassic shallow‐water limestone (Calcare Massiccio, cf. Table 1).

With respect to sediment input, the Campo Imperatore basin shows a distinct asymmetry. Except for the westernmost tip of the basin (Mt. Aquila to Mti. della Scindarella), no sizeable sediment input from alluvial fans or large scree slopes takes place from the southern flank that, instead, is dominated by karstic denudation and fluviokarstic processes (Figs 1B and 2). At present, sediment input into the basin is practically exclusively conveyed by large active alluvial fans that all are supplied by catchments incised into the northern front range (Figs 1B, 1C and 2). In the study area, the alluvial fan supplied by the Fornaca catchment extends across the whole width of the basin. Near the southern basin margin, however, the Fornaca fan onlaps a fault‐bounded horst (Fig. 1C). From bottom to top, this horst exposes a Pleistocene succession that comprises: (i) an interval of cemented alluvial conglomerate (‘Old Conglomerate’) that unconformably overlies truncated Mesozoic rocks; (ii) the cemented rock‐avalanche deposit (in the following dubbed ‘Rubble Breccia’) dealt with in more detail herein; and (iii) unlithified alluvial‐fan deposits perched above the present level of the Fornaca fan (Figs 2 and 3; Table 2).

**Table 2:** General characterization of exposed succession in the horst bounded by the Cucchiarelle fault system, from youngest (top) to oldest (bottom). Names in inverted commas are informal. See also Fig. 3.

Designation	Characterization	Interpretation
‘Perched Fan Deposit’	Unlithified pebbly deposit with a matrix of sand and, locally, with a few cobbles; pebbles to cobbles are subrounded to well‐rounded and consist of the same lithological spectrum as present along the northern margin of the Campo Imperatore basin (cf. Table 1). Age: Pleistocene	Alluvial fan deposit
‘Rubble Breccia’	Unstratified, extremely‐poorly sorted deposit of angular clasts up to boulder size; ‘crackle’ and ‘jigsaw’ fabrics are common (Fig. 4). See text for more detailed description. Age: Pleistocene	Rock avalanche deposit
‘Old Conglomerate’	Parallel‐stratified and more rarely cross‐stratified, poorly to well‐sorted, fine to coarse‐pebbly conglomerates. Clast fabrics of a(t),b(p,i)‐type common. Age: Pleistocene	Lithified deposit of an ephemerally‐active braided stream
Calcare Massiccio	See Table 1 for characterization. Age: Hettangian to Sinemurian	Shallow‐water limestones

Today, surface runoff in the Campo Imperatore basin is highly ephemeral, and directed towards the east. At the eastern end of the basin, a bedrock gorge incised down to approximately 300 m in depth of the Tavo stream is not associated with a mappable alluvial fan at its debouch (Fig. 2). The gorge and its tributaries are dry over most of the year. Similarly, surface runoff on the alluvial fans is highly ephemeral. Evidence for a formerly higher discharge, however, is provided by: (i) abandoned and vegetated spring pits and channels on the fans; (ii) rivulets that today are abandoned, and that are incised into the fault‐bounded horst (see below for further description); and (iii) incision of wide, vegetated, flat‐floored channels presumably underlain by formerly active braided streams into all of the alluvial fans of the eastern Campo Imperatore basin. Precisely when this phase of increased discharge occurred, however, as yet is elusive.

## METHODS, DEFINITIONS

The deposit that is the focus of this study, i.e. the Rubble Breccia (RB), and the succession it is part of, were mapped in several field campaigns from 2016 to 2019. Mapping was based on the topographic map 1:25.000 Gran Sasso d'Italia and on satellite orthoimages provided by Google Maps^®^. The RB is positioned subhorizontally and was not subject to significant post‐depositional tilting. The thickness of the RB was determined by field mapping in satellite orthoimages followed by altitude measurement with 1 m isohypse steps of the mapped interval in Google Earth^®^. Because the altitude step in the satellite data is 1 m, the imprecision of these thickness measurements is less than 1 m. This method, supported by outcrop overviews photographed in the field, yielded the best results along the steep, well‐exposed flanks of the Valle Cortina canyon and of an incised ravine directly west of Colle Caciaro (Fig. 3).

A total of 12 samples of the RB were studied in cut and polished slabs as well as in thin section. The samples were taken from disparate locations across the deposit (Fig. 3). In the field, in the RB, it typically was only possible to distinguish between clasts of limestone and dolostone, but limestone facies could not assessed with sufficient certainty. A more precise assessment of clast spectrum of the RB was based on 11 thin sections 4 × 6 cm in size (see Data S1). In addition, seven thin sections of the Old Conglomerate below the RB were used for a comparison of clast spectra of both stratigraphic intervals (see Data S2).

A sample (CI 60) of coarse‐sparry calcite cement (see below for further description) obtained from within the RB was dated using U/Th disequilibrium dating. Six powdered sub‐samples of 30 to 225 mg were collected from a cut slab of the CI 60 cement using a scalpel and microdrill. Chemical treatments and mass spectrometric measurements were carried out in the Isotope Geochemistry Laboratory at the University of Minnesota. Separation and purification of U and Th aliquots was achieved using standard methods (Edwards *et al*., 1987) in a clean air environment. Samples were spiked with a dilute ^229^Th–^233^U–^236^U tracer to allow for correction of instrumental fractionation and calculation of U and Th concentrations and ratios. Uranium and thorium isotopes were measured using a ThermoFinnigan Neptune mass spectrometer (Thermo Fisher Scientific, Waltham, MA, USA), using the instrumental procedure according to Shen *et al*. (2012). Data reduction and correction for mass fractionation, abundance sensitivity, background intensities, nonlinearity, yield, spike and chemical blanks were performed offline. Due to high detrital Th concentrations (Table S3), the results were further analysed using an isochron in order to determine the initial ^230^Th/^232^Th ratio and corrected age (Data S4; Ludwig & Titterington, 1994; Dorale *et al*., 2004).

**Fig. 4:**
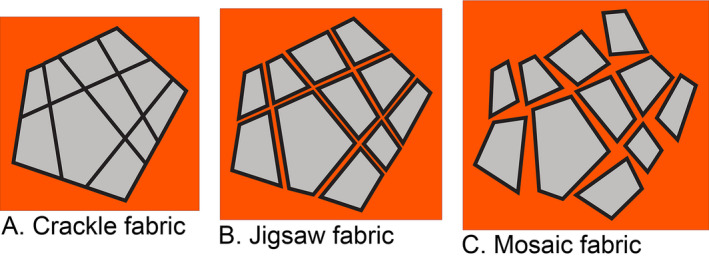
Scheme to illustrate designation of fabrics that originated by disintegration of a larger precursor clast into smaller subclasts while embedded in a surrounding medium (comminuted rock or sediment). For further characterization see text and Table 3.

Herein, the designation of grain sizes follows the extended Wentworth (1922) scale of Blair & McPherson (1999) for a subdivision of size classes larger than 256 mm in mean diameter. The designation of clast shape axes follows Krumbein (1941), with the a‐axis the longest, the b‐axis the intermediate and the c‐axis the shortest. The notation of clast fabrics is according to Collinson & Thompson (1989). For the present purpose, a rock avalanche is understood as a gravity‐driven, extremely rapidly propagating mass‐wasting of rock with a volume of at least 10^5^ m^3^ that undergoes dynamic fracturation during transport (see above) until it *comes to rest as a rock avalanche deposit at a site of later observation*. Dealing with incompletely preserved and/or patchily exposed fossil rock avalanches, the latter specification is necessary to exclude *debris* avalanches from consideration, although these may develop from initial rock avalanches (cf. Hungr & Evans, 2004; Dufresne & Geertsema, 2020). Fossil debris avalanches are not dealt with herein. To designate the fabrics of fragmented rock masses, a combined Sibson (1977)–Heitzmann (1985) terminology is used that chiefly distinguishes between: (i) lithified fragmentites or cataclasites; and (ii) unlithified comminuted rock or cacirite (Sanders *et al*., 2018a, fig. 4). To designate the fabrics that originate by fracturation of a larger clast while it is embedded within a surrounding sediment or comminuted rock, and to designate fabrics that show no organization with respect to clast orientation and size, terms as outlined in Table 3 and illustrated in Fig. 4 are used.

**Table 3:** Designation of fabrics that: (i) originate by fracturation of a larger clast while it is embedded within a surrounding sedimentary matrix or comminuted rock; and (ii) for an unstructured mass of fragmented rock not originated from a single precursor clast.

Fabric type	Characterization	Remarks, references
Crackle fabric (see Fig. 4)	Fragmentation by densely‐spaced swarms of fractures of different orientations; subclasts are only minimally removed from one another; fitting subclast boundaries easy to see	Phillips (1972), Sibson (1986), Woodcock & Mort (2008), Melosh *et al*. (2014) One fracture set may prevail (cf. Melosh *et al*., 2014)
Jigsaw fabric (see Fig. 4)	Fragmentation by densely‐spaced swarms of fractures of different orientations; subclasts are slightly and consistently removed from one another, resulting in an ‘exploded’ appearance; interstitial space may be filled with diagenetic minerals or fine‐grained cataclastic matrix	Phillips (1972), Sibson (1986), Tarasewicz *et al*. (2005), Melosh *et al*. (2014), Dufresne *et al*. (2016)
Mosaic fabric (see Fig. 4)	Fitted subclast boundaries can still be identified, but (at least in larger patches) subclasts are significantly translated and rotated relative to one another, resulting in a wider interstitial space that may be filled with diagenetic minerals or fine‐grained cataclastic matrix	Depending on amount of local strain, transitions from crackle fabric via jigsaw to mosaic fabric are typical Woodcock & Mort (2008), Melosh *et al*. (2014)
Disordered fabric	Clast boundaries do not correspond with one another; fabric comprises different lithologies not derived from a single larger precursor clast; clasts do not show a preferred orientation; no normal/inverse grading, no clast imbrication	Corresponds to the ‘chaotic fabric’ of Woodcock & Mort (2008) and Melosh *et al*. (2014)

## DESCRIPTION

### Stratigraphic context

Along its distal southern margin, the Fornaca alluvial fan onlaps a horst bounded by the Cucchiarelle fault system (Figs 1C, 2 and 3). At fault oversteps, the present drainage of the Fornaca fan traverses the horst along a bedrock‐incised canyon floored with an ephemerally‐active braided stream (Fig. 3). From bottom to top, the exposed horst succession consists of: (i) Calcare Massiccio (see Table 1); (ii) a succession of Quaternary alluvial conglomerates (‘Old Conglomerate’); (iii) the RB described herein; and, at the top, (iv) unlithified alluvial fan deposits that became perched in an elevated position due to faulting (‘Perched Fan deposit’) (Fig. 3, Table 2). The RB overlies a composite unconformity cut mainly into the Old Conglomerate and, on Colle Caciaro (Fig. 3), locally also into the older bedrock. All of the Pleistocene depositional units are involved in faulting (Fig. 3).

Over much of its extent, the RB overlies the Old Conglomerate (Fig. 3). Near the Cucchiarelle fault system, in contrast, the RB is overlain by the Perched Fan deposits. The stratigraphic contact of the RB with both the underlying Old Conglomerate and the overlying Perched Fan deposits is preserved only in an outcrop along the right flank of the Valle Cortina canyon. There, altitude‐based thickness measurement (see section *Methods*) yielded a thickness of the RB of 17 m (Fig. 3). On the opposite, left canyon flank, also a thickness of 17 m is determined to the preserved top of the RB. On the western flank Colle Caciaro (Fig. 3), two measurements from the top of the Old Conglomerate to the preserved top of the RB in each case yielded a preserved thickness of the RB of 13 m. At other locations, determinations of thickness yielded slightly lower figures.

**Fig. 5:**
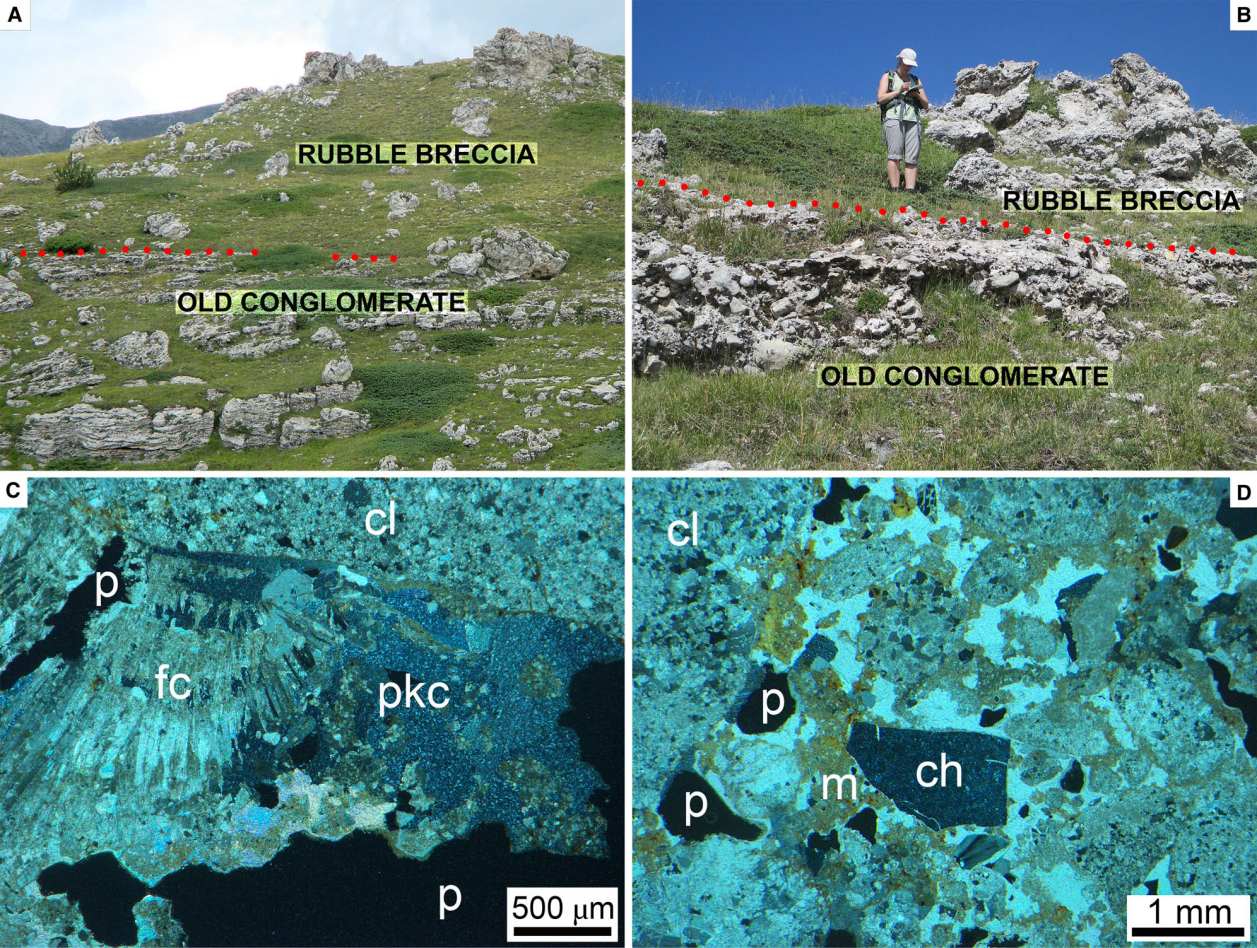
Stratigraphic contact between the Old Conglomerate and the overlying Rubble Breccia on the western flank of Colle Caciaro (cf. Fig. 3). (A) and (B) The vertical contact is a sharply defined surface (red stipples) on top of the Old Conglomerate. Width of view in (A) *ca* 30 m; person for scale in (B) is *ca* 1.8 m tall. (C) and (D) Thin section photographs of a sample from the top of the Old Conglomerate shown in (B). (C) Below a carbonate lithoclast (cl), fibrous calcite cement (fc) had grown that shows relicts of successive growth phases (short white lines). This fibrous cement became partly replaced by a poikilotopic cement (pkc) rich in inclusions. The entire fabric is cross‐cut by dissolution pores (p). Crossed nicols. (D) Conglomerate mainly of carbonate lithoclasts (one labelled ‘cl’) and a few clasts of chert (ch), and with remnants of brownish matrix (m), is cemented by poikilotopic calcite spar (=whitish area between clasts). The entire fabric is cross‐cut by dissolution pores (p). Crossed nicols.

The continuous exposures in the cliffs along the Valle Cortina canyon and its tributaries indicate that the Old Conglomerate was not subject to soft‐sediment deformation (for example, drag folding, shear‐out of rafts) by the overriding rock avalanche. At Colle Caciaro, the well‐exposed contact between the Old Conglomerate and the RB is sharp and concordant with undeformed bedding in the conglomerate (Fig. 5A and B). At Colle Caciaro, because of onlap onto a relief of the bedrock, the Old Conglomerate locally pinches out, and the RB directly overlies the older bedrock (Fig. 3).

In thin section, the topmost bed of the Old Conglomerate sampled at the location of Fig. 5B displays a high amount of interstitial cement, whereby a radial‐fibrous calcite spar became more‐or‐less completely replaced by a poikilotopic calcite spar (Fig. 5C). Alternatively, except for younger dissolution pores, the entire pore space is filled by poikilotopic calcite spar (Fig. 5D). In the RB directly above this surface, in contrast, cement is as sparse as nearly everywhere in this deposit (see below).

### Fabrics

**Fig. 6:**
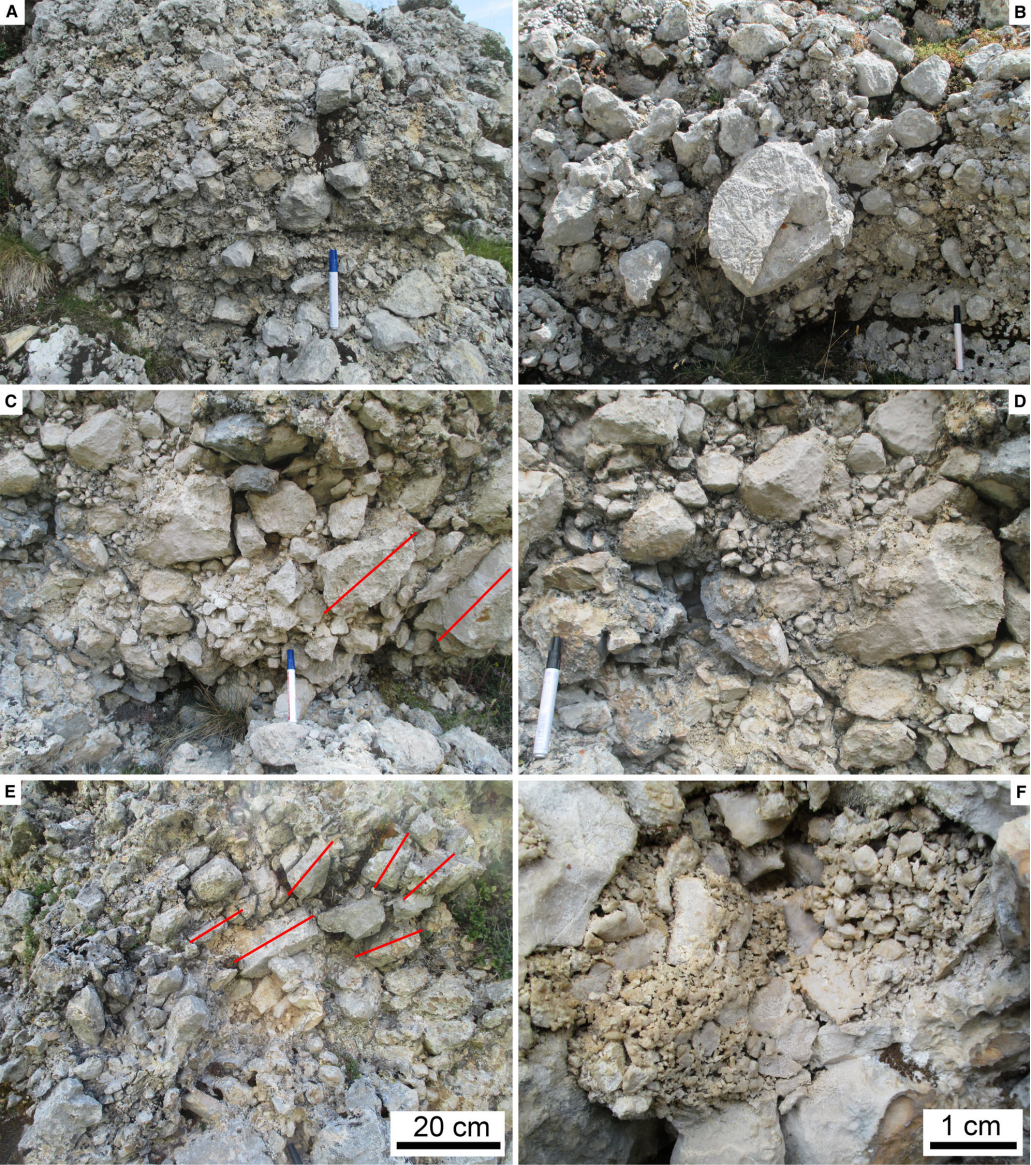
Fabric of Rubble Breccia. (A) to (E) Metre to decimetre‐scale appearance in the field, displaying an extremely‐poorly sorted, tightly packed to interlocked fabric of angular to subangular clasts. Subfigures (C) and (E): note single clasts and arrays of clasts embedded with their a,b‐axial planes steeply inclined (thin red lines). Pen is 14 cm in length. (F) Porous matrix of sand to fine‐pebble sized angular rock fragments.

**Fig. 7:**
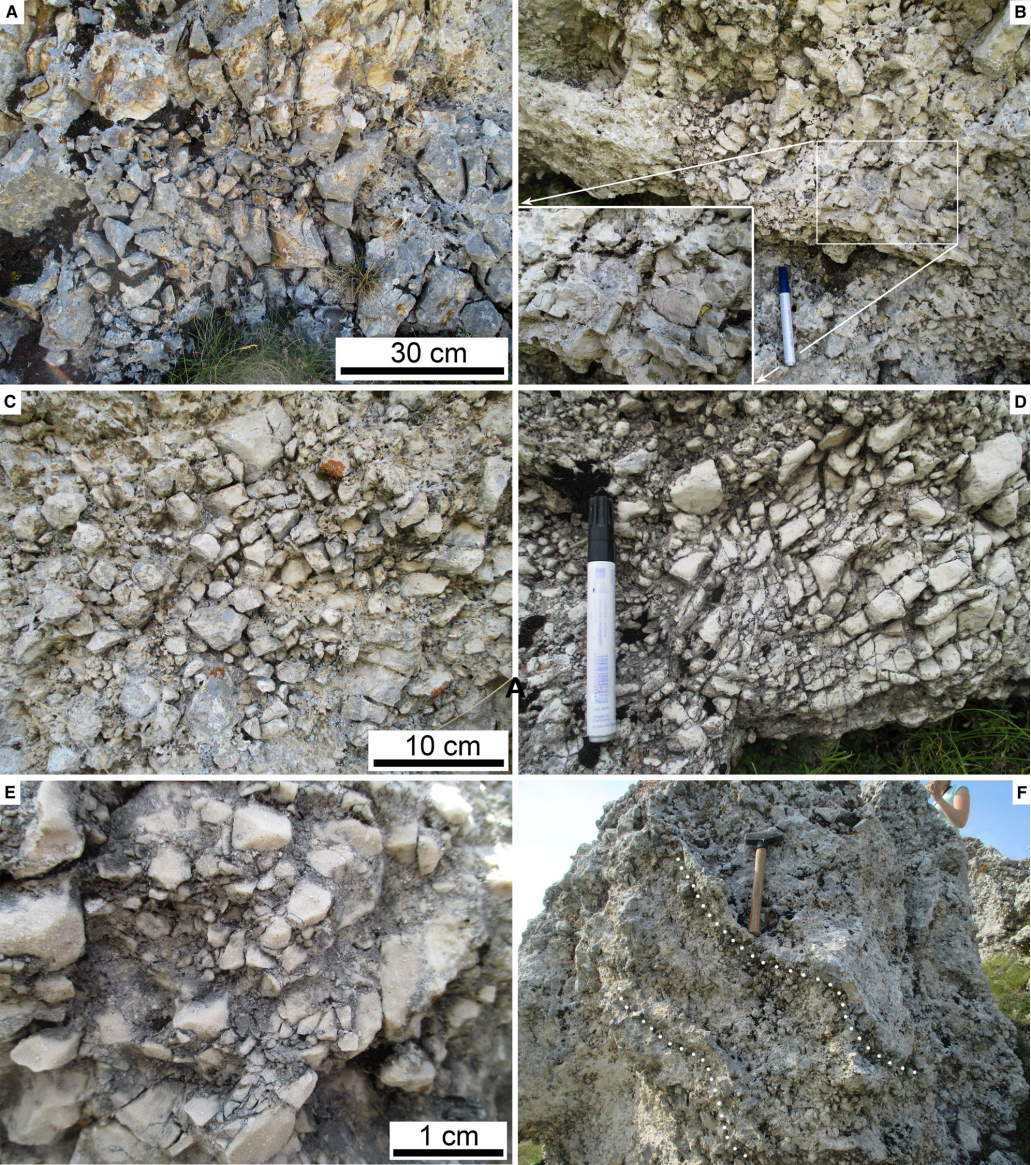
Fabric of Rubble Breccia, continued. (A) Boulder disintegrated to jigsaw breccia (see Table 2 for definition of fabrics). (B) Detail of boulder fragmented to a crackle to jigsaw breccia (inset). Pen: 14 cm. (C) and (D) Cobbles disintegrated to jigsaw breccia in their central part and grading into a mosaic breccia with progressively more translated/rotated subclasts in their marginal part. (E) Pebble crushed to a crackle fabric. (F) Outweathered, tilted and slightly folded shear belts (base stippled white). Hammer is 33 cm in length.

**Fig. 8:**
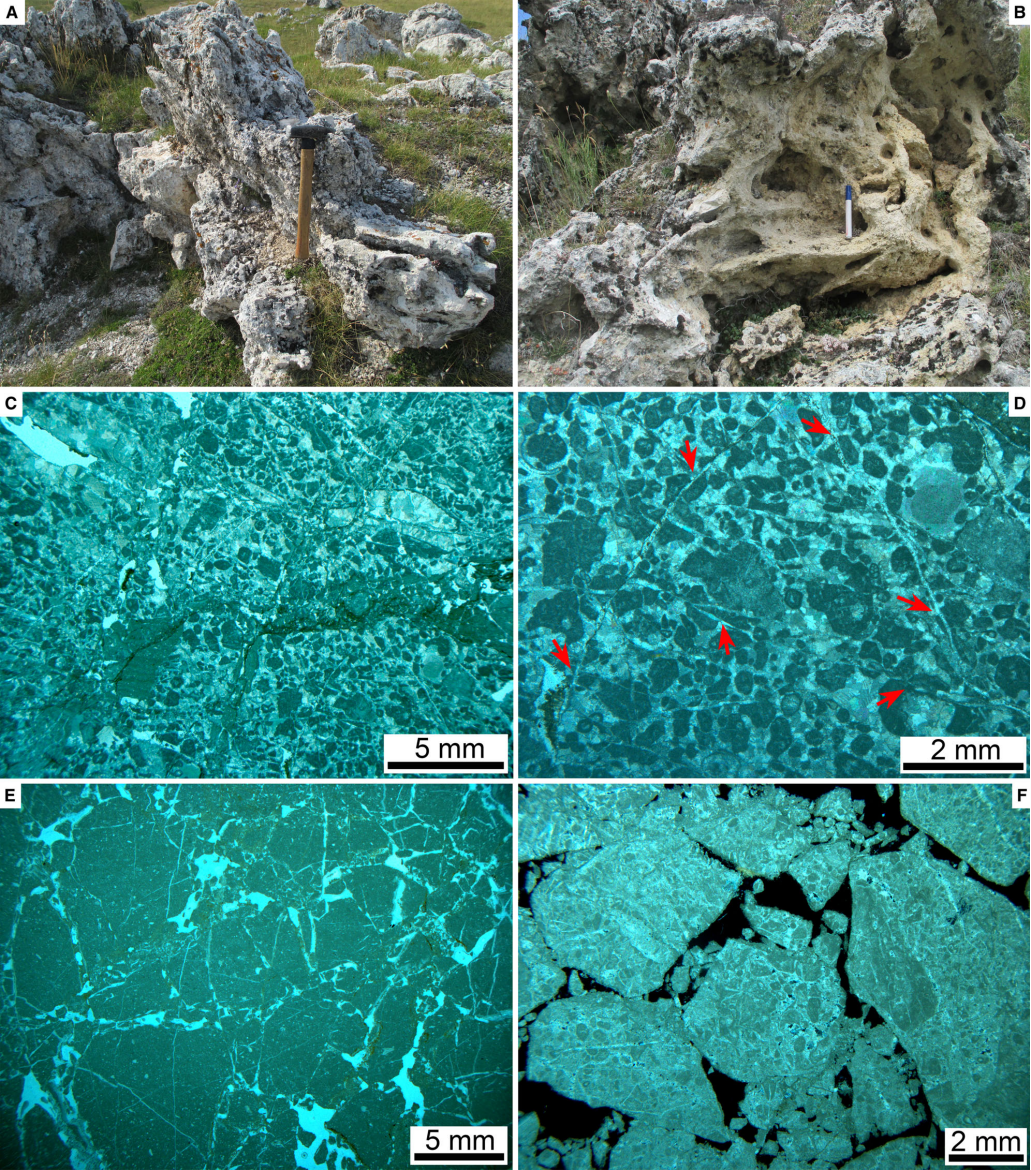
Fabric of Rubble Breccia, continued. (A) Outweathered, steep‐tilted shear belts. Hammer is 33 cm in length. (B) Folded outweathered shear belts. Pen: 14 cm. (C) and (D) Crackle fabric of apparently intact domain of shallow‐water bioclastic grainstone (C) in detail reveals numerous fractures associated with small offset indicated by sediment components (some fractures indicated by red arrows in D). Parallel nicols. (E) Jigsaw fabric of deep‐water wackestone with *Saccocoma* ossicles. Fine‐grained cataclastic matrix originally present between subclasts (arrows) has been partly dissolved away (light patches). Parallel nicols. (F) Mosaic fabric of shallow‐water grainstone. The scarcity of interstitial matrix may result from nearly wholesale dissolution. Crossed nicols.

In the field, the RB mainly appears as a disordered, extremely‐poorly sorted mass of angular to subangular clasts up to boulder size grade (Fig. 6A to C). Locally, clasts are oriented with the a‐axis dipping towards the northern sector (north‐east – north‐west) (Fig. 6C and E). Matrix is scarce (Fig. 6C and D), and the interstitial space between larger clasts is filled with a fine‐grained ‘rubble’ of angular clasts of the same lithology as are the larger clasts (Fig. 6F). At many locations, domains between *ca* 1 m to a few millimetres in width are seen that internally consist of angular clasts (of the same lithology) with fitted boundaries (Fig. 7A to E). Along their margins, these domains typically are gradational, and their constituting subclasts are more‐or‐less translated and rotated out into the surrounding deposit, whereas the core of these domains commonly shows tighter‐packed and better fitted subclasts (Fig. 7A to D). Another feature observed in particular on Colle Caciaro (cf. Fig. 3) is the presence of planar features weathered proud of the breccia mass (Figs 7F, 8A and 8B). These planar features are more‐or‐less inclined and folded and, internally, consist of relatively fine‐grained cataclasite with an ultracataclastic groundmass.

**Fig. 9:**
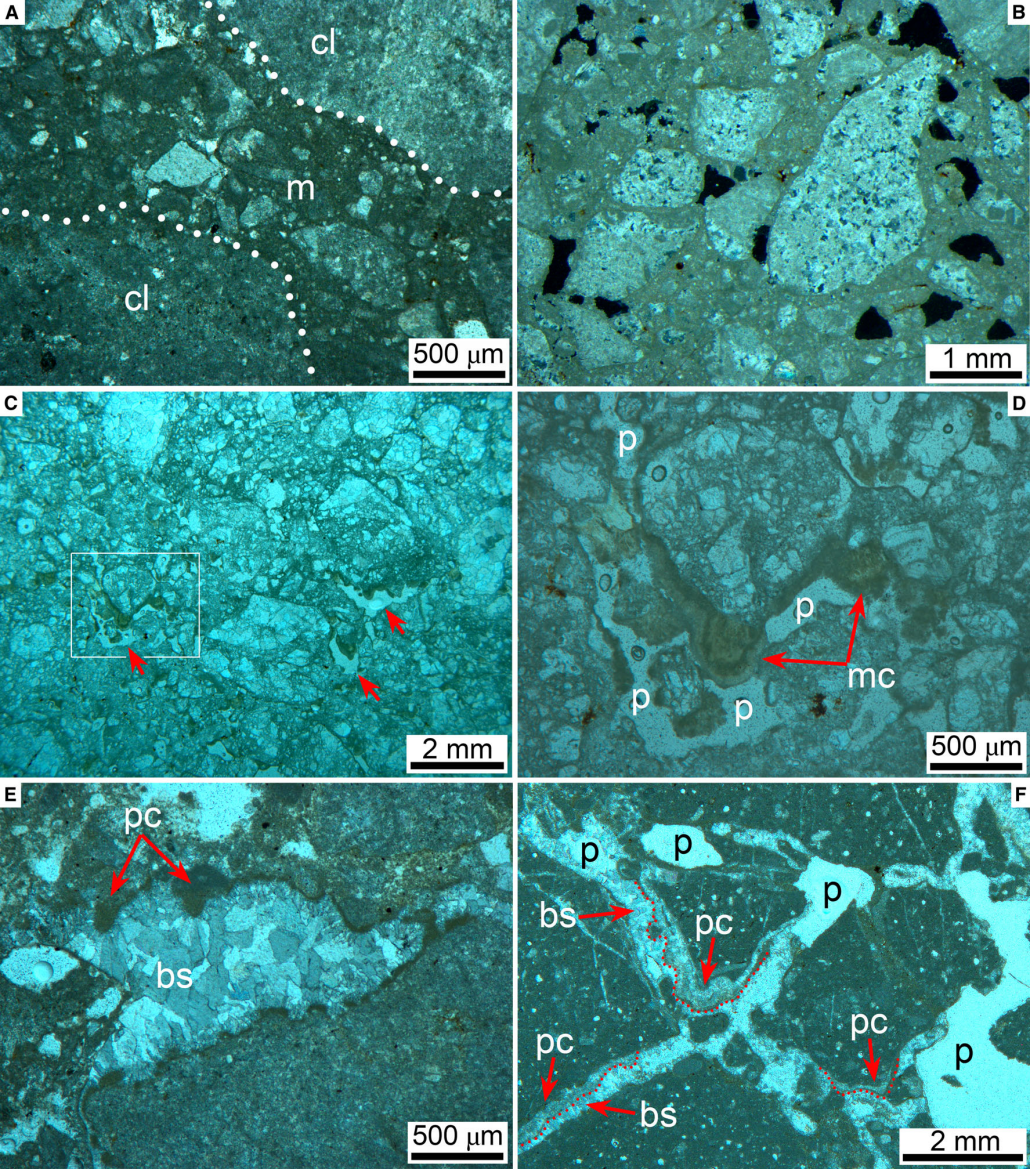
Fabric of Rubble Breccia, continued. (A) and (B) Thin sections from domains relatively rich in interstitial matrix (‘m’) between clasts (‘cl’, outline stippled white). In (B), the matrix between clasts of dolostones (‘d’) is riddled with dissolution pores (black patches). (A): parallel nicols; (B): crossed nicols. (C) and (D) Sample from a larger dolostone domain in the Rubble Breccia. Whereas most of the original cataclastic matrix is preserved (upper part of subfigure C), it is locally cross‐cut by dissolution pores (red arrows in C, labelled ‘p’ in D) that are decorated with microstalactitic cement (‘mc’ in subfigure D). White rectangle in C delimits area shown in subfigure D. Parallel nicols. (E) Dissolution pore fringed with; (i) mammillary and pendant micritic cement (labelled ‘pc’), followed by (ii) blocky calcite spar (labelled ‘bs’) that fills the remaining pore space. Parallel nicols. (F) Fracture pores overprinted by dissolution; followed by (i) precipitation of pendant cements (‘pc’, outlined by red stipples) and (ii) by blocky calcite spar (‘bs’) that filled the remaining pore space. Note also pores widened by later dissolution (white patches labelled ‘p’) that cross‐cut all older fabrics. Parallel nicols.

In thin section, larger clasts that appear relatively intact in the field are seen to be cut by numerous fractures with an offset in the submillimetre to millimetre range (Fig. 8C and D). Preserved matrix content and translation/rotation of fracture clasts relative to one another are highly variable, but matrix in general is scarce (Fig. 8E and F). Only locally, in patches, interstitial fractures and pores are filled with ultracataclastic matrix (Figs 8E, 9A and 9B).

### Clast spectrum

With respect to clast lithologies, in its limestone‐dominated portions, the RB mainly consists of: (i) Mesozoic shallow‐water bioclastic limestones (for example, Fig. 8C, D and F); (ii) coarse‐crystalline dolostones (Fig. 9B to D); and (iii) lime mudstones to bioclastic wacke–packstones with fine‐grained crinoid debris (probably of *Saccocoma*) and hyaline smaller benthic foraminifera (Fig. 8E) (see Data S1). In the underlying Old Conglomerate, the clast spectrum is consistently dominated by typically coarse‐crystalline dolostones. In addition, a wide spectrum of limestones is present, such as oolithic grainstones and shallow‐water bioclastic/peloidal grainstones to packstones to wackestones, deep‐water limestones with microfossils that indicate different ages ranging from the Early Cretaceous to the Cenozoic, protocataclasites and cataclasites of dolostones or limestones, and a low but constant amount of chert (see Data S2). Cursory observation of the sediment of the *presently* active Fornaca alluvial fan, in turn, indicates that it is strongly dominated by clasts derived from the Upper Triassic dolostone succession exposed in the Fornaca catchment in the range front (Figs 1C and 2).

### Diagenesis

**Fig. 10:**
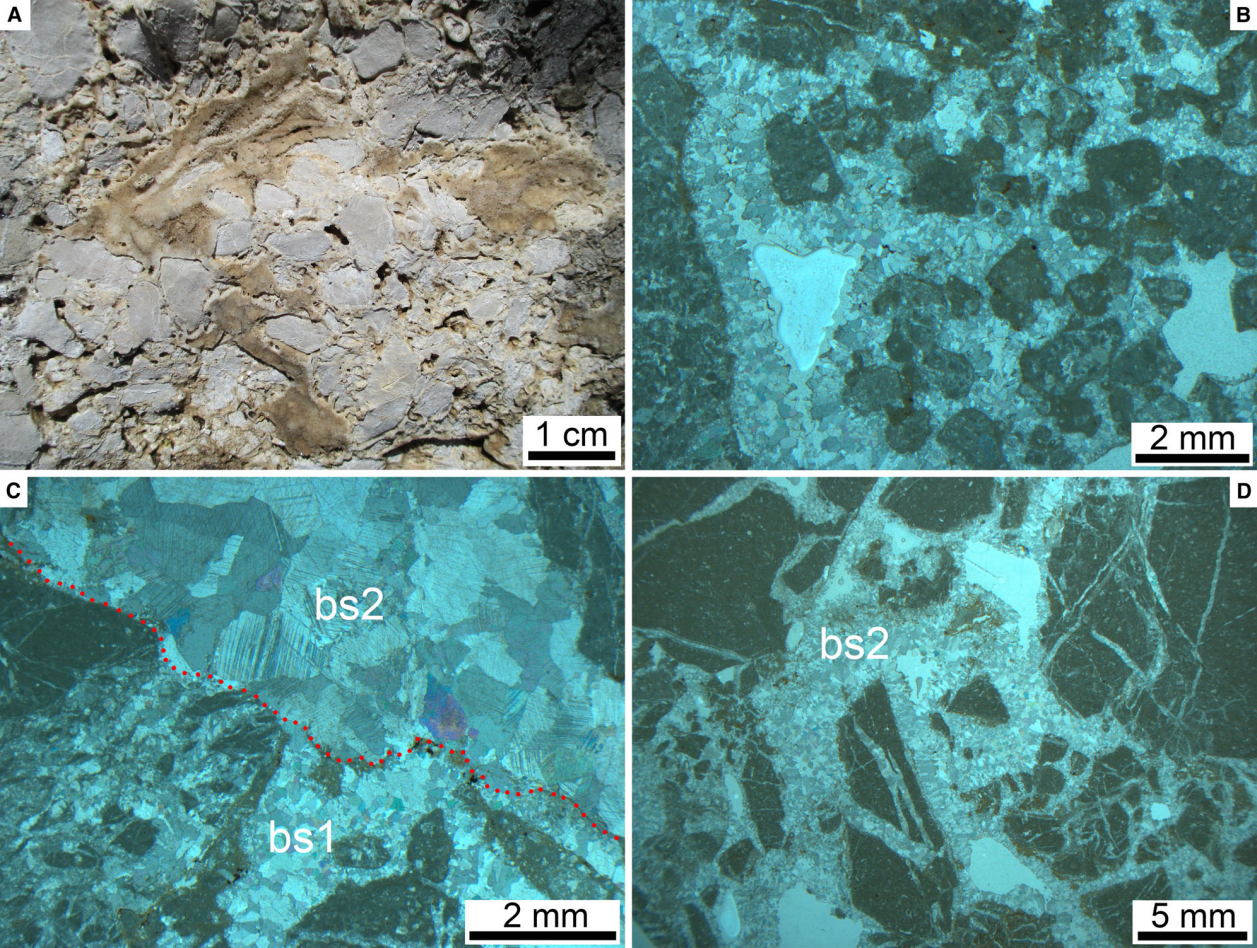
Cement used for U/Th dating. (A) Fracture surface in Rubble Breccia patchily coated by brownish calcite cement. (B) Dissolution clasts (note pitted and embayed outline of clasts) of cataclastic matrix and rock fragments coated by an isopachous fringe of calcite cement. Parallel nicols. (C) A first phase of dissolution followed by blocky calcite spar (bs1) was again followed by dissolution (red stipples) and precipitation of coarse sparry cement (bs2). This coarse sparry cement bs2 was microdrilled for U/Th dating. Parallel nicols. (D) The precipitation of sparry cement bs2 was preceded by fracturation. Parallel nicols.

Records of diagenetic overprint of the RB include highly patchy preservation of interstitial ultracataclastic matrix, mouldic pores (Fig. 9B and C), microstalactitic and mammillary cements (Fig. 9D and E) and fracture porosity that has been filled, or partly so, by pendant cement and/or by isopachous fringes and small pore fillings of calcite spar; in addition, late‐stage dissolution of lithoclasts and cement, and dissolution‐induced widening of pores is common (Fig. 9E and F). Within the RB, well‐preserved calcite cement is very rare. At a single location labelled U/Th in Fig. 3, however, a domain with fringes of calcite cement thick enough to be sampled with a microdrill for U/Th dating has been found (Fig. 10A). At this site, the fabric shows evidence for repeated phases of dissolution, fracturation and cement precipitation (Fig. 10B to D). Attempts were made to only sample the coarse‐sparry calcite cement that, according to petrography, represents the youngest phase of cement (Fig. 10A, C and D). Of six sub‐samples taken from this cement within sample CI 60, one was open‐system, and another one was too high in detrital Th to be analysed. The other four sub‐samples yielded results that were further interpreted using an isochron (Dorale *et al*., 2004), indicating an elevated initial ^230^Th/^232^Th ratio of *ca* 20 ppm and an age of 124.25 ± 2.76 ka bp (1950) (see Data S3 and S4). Given the repeated phases of dissolution/cementation, as evidenced by the cement fabric, this figure has to be considered as an *ante‐quam* age that post‐dates the mass‐wasting event by an unknown lapse of time.

## INTERPRETATION AND DISCUSSION

### General features of rock avalanches

In previous geological maps, the RB has been ascribed to reworked glacial moraines (Alberti *et al*., 1963) or to poorly‐sorted pebbly alluvial deposits (Vezzani & Ghisetti, 1998). Inasmuch, both of these deposit types tend to be poorly to extremely‐poorly sorted, or contain extremely‐poorly sorted intervals at least, this seems understandable. The RB, however, shows features incompatible with an origin from debris flows and moraines. Before proceeding to details of distinction, a brief account on the propagation and the deposits of rock avalanches is indicated. Provided unconfined runout, rock avalanches can propagate long distances over horizontal terrain (Heim, 1932; Howard, 1973; Hsü, 1975, and many others). This ‘excessive’ runout requires that the dynamic frictional resistance of rock avalanches is much smaller than the static frictional resistance of the same blocky material. Hypotheses to explain this may be grouped into: (1) lubrication of the moving mass by external materials such as trapped air, snow, ice, water, water‐logged overridden sediment, and water vapour derived from frictional heating of pore water (e.g. Kent, 1966; Perinotto *et al*., 2015; Watkins *et al*., 2015; Dufresne *et al*., 2016); and (2) explanations that hinge on features of the moving mass itself, such as ‘self‐lubrication’ due to: (i) the mechanics of moving granular masses (Campbell, 1989, 2006; Blasio, 2011; Schilirò *et al*., 2019); (ii) dynamic disintegration of rock (e.g. McSaveney & Davies, 2002, 2009; Imre *et al*., 2010; Zhang & McSaveney, 2017; Davies *et al*., 2020); (iii) frictional weakening/heating and thermal pressure buildup along the base (Borykov *et al*., 2019; Liu *et al*., 2019); (iv) dissociation of carbonate minerals upon frictional heating, combined with superheated steam (Mitchell *et al*., 2015; Hu *et al*., 2019); and (v) acoustic fluidization (Cruden & Hungr, 1986; Collins & Melosh, 2003). None of these concepts are mutually exclusive, and combinations of external and internal amplifiers of rock‐avalanche mobility are common (Hungr & Evans, 2004; Zhao *et al*., 2018).

In vertical section, from top to bottom, completely preserved RADs display: (i) a carapace rich in boulders and blocks; (ii) a main mass, or body, of an extremely‐poorly sorted mixture of clasts ranging in grain size from nanometer‐scale particles to boulders, and with gradationally‐bound lenses rich in cobbles to blocks with crackle to mosaic fabric (cf. Table 3, Fig. 4); in addition, cataclastic shear belts richer in fine‐grained matrix are typical; and (iii) a basal interval of relatively fine‐grained comminuted rock (Dufresne *et al*., 2015, 2016; Wang *et al*., 2018, 2019, 2020; Zeng *et al*., 2019). Densely‐fragmented cobbles to blocks with crackle to mosaic fabric originate by fracture of previously larger clasts upon mutual high‐energy collisions during rock‐avalanche propagation (Davies & McSaveney, 2002, 2009; Imre *et al*., 2010; Perinotto *et al*., 2015; Dufresne *et al*., 2016; Wang *et al*., 2019). Despite the mentioned differences to explain rock avalanche runout, there seems to exist broad consensus that masses propagated as rock avalanches *sensu stricto* always display dense fragmentation of many of their constituent larger clasts. To distinguish RADs – be they recent or fossil – from other deposits, thus, the two most diagnostic features are: (i) densely‐spaced fragmentation of apparently intact blocks; and (ii) common presence of clasts with crackle to mosaic fabric that obviously originated from a single larger clast that had been disintegrated into smaller subclasts during transport (Figs 7A to E and 8C to F).

### Interpretation of the Rubble Breccia

The preserved thickness of the RB of approximately 10 to 17 m would fit well with the distal parts of post‐glacial rock avalanches, which typically are between 10 m to a few tens of metres in thickness (e.g. Patzelt, 2012a,b; Dufresne *et al*., 2016). In the present context, however, thickness is of less relevance, because: (i) the RB is topped by an unconformity; and (ii) the intention of the present paper is to determine how to identify fossil rock‐avalanche deposits that are *no* longer obvious by surface morphology and complete preservation, be it due to erosion and/or to burial by other deposits. As outlined above, the ‘body’ of rock avalanche deposits is characterized by jigsaw and mosaic clast fabrics, whereas the shallow level of rock avalanches is an extremely‐poorly sorted carapace rich in pebble to block‐sized clasts that, however, do not show jigsaw and mosaic fabric. The observation that the RB shows jigsaw and mosaic fabrics up to its preserved top thus underscores that an unknown portion of the original rock avalanche deposit has been eroded before it became overstepped by the Perched Fan deposits.

**Fig. 11:**
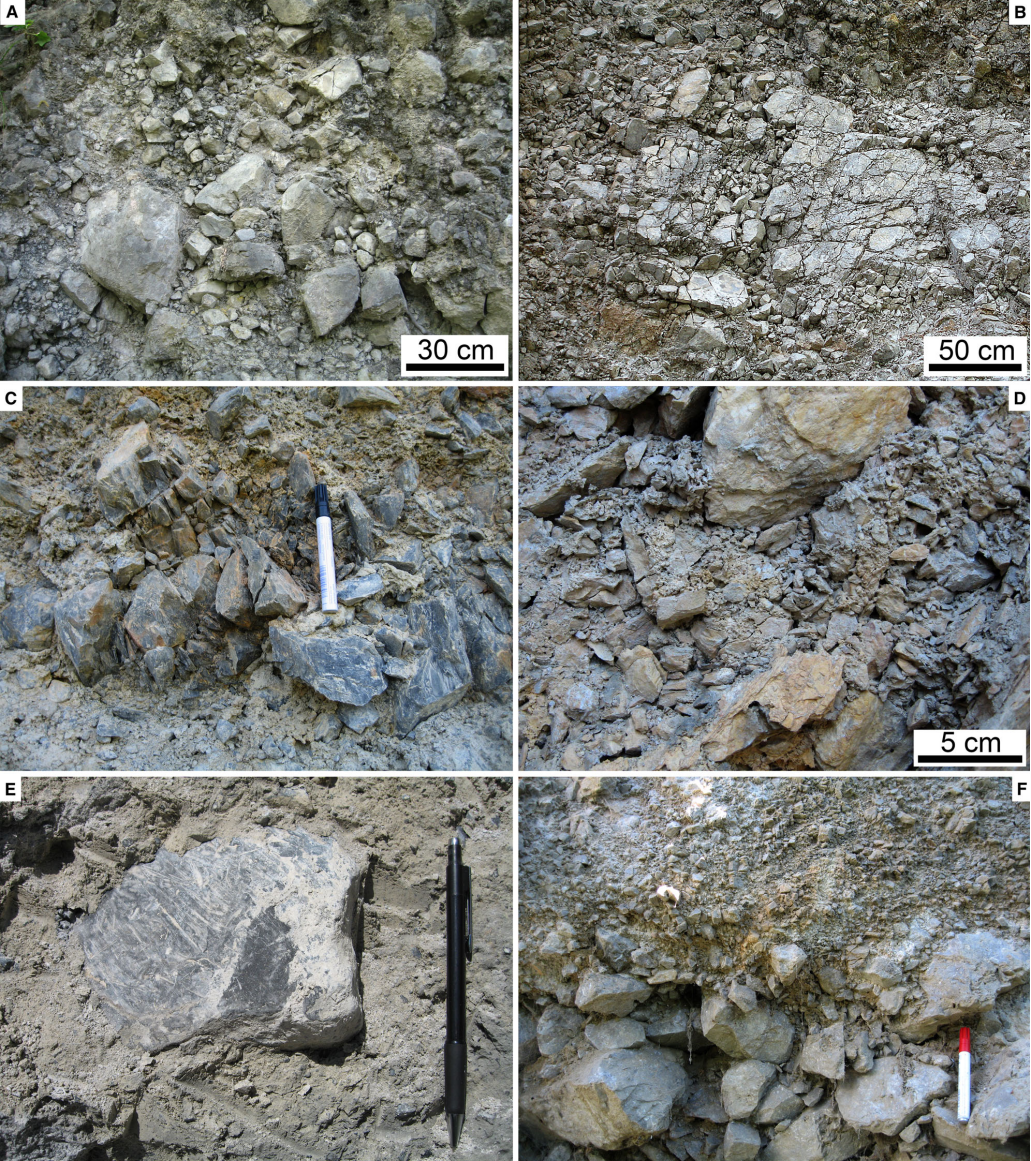
Comparative fabrics of young (post‐last glacial) rock avalanches (see Data S5 for additional information). (A) Extremely‐poorly sorted, disordered fabric of angular clasts of sand to boulder size; cf. Fig. 5A to C. (B) Boulder patchily disintegrated into domains of crackle breccia and domains of jigsaw to mosaic breccia; cf. Fig. 6A to E. (A) and (B): Marocche di Dro, Southern Alps. (C) Disintegration of a boulder embedded in finer‐grained lithoclastic material. Pen: 14 cm; cf. Fig. 6C (D) ‘Rubbly’ matrix consisting of angular, sand to fine pebble‐sized fragments of the same lithology than the larger clasts. Pen: 14 cm. cf. Fig. 5F. (E) Striated clast in an ultracaciritic shear belt. Pen: 14.5 cm. (C) to (E): Tamins, Western Alps. (F) Domains of highly different degrees of rock disintegration. Pen: 14 cm. Tschirgant, Eastern Alps.

A comparison of the fabrics of young (= post‐glacial) rock avalanches (Fig. 11A to D) with the fabrics observed in the RB (Figs 6 and 7) supports an interpretation of the latter in terms of a rock avalanche deposit (see Data S5 for locations and references of comparative examples). In particular, the common presence of cobbles to blocks with a crackle, jigsaw to mosaic fabric is considered diagnostic of catastrophic mass‐wasting rather than of processes related to debris flows or subglacial deposition. Similarly, a matrix of an unsorted rubble of angular, silt to pebble‐sized fragments of rock of identical lithology to the larger clasts (compare Fig. 6F with Fig. 11D) seems to be a good supportive indicator of a rock‐avalanche origin. From Table 4, it is evident that deposits of debris flows, tills and rock avalanches share some characteristics, in particular if taking into account the natural variability of deposits. For instance, polished and striated clasts embedded in compact ultracacirite along shear belts in rock avalanches may look very similar to glacially‐polished/striated clasts (Fig. 11E). In RADs, in particular in the case of limited outcrop, marked vertical differences in the degree of rock comminution may be confused with stratification of debris‐flow deposits (Fig. 11F). Furthermore, glacitectonites may appear broadly similar to fabrics near the base of rock avalanches that had entrained foreign material (compare Evans *et al*., 2006, fig. 26, and Aber & Ber, 2007, with Patzelt, 2012b, and Dufresne *et al*., 2016).

**Table 4:** Summary of features of rock avalanche deposits, and differential diagnosis with respect to cohesive debris flows and subglacial diamicton. See text for further discussion

	Cohesive debris flow	Subglacial diamicton	Rock avalanche
Internal organization	Overall disordered clast fabric, moderately to extremely poorly sorted (depending on source material), but blocks are rare; clast to matrix‐supported; may show inverse or normal grading; may display updip or downdip imbrication of clast a‐axes; depositional lobes may show distinct downdip a‐axis imbrication of clasts. In detail, imbrication direction and style (a‐axis or b‐axis imbrication) highly variable within an individual event deposit Eyles & Kocsis (1988), Coussot & Meunier (1996), Major (1997, 2000)	Disordered clast fabric to strongly preferred orientation of clast a‐axes parallel to ice flow; typically matrix‐supported; may contain more or less deformed rafts of other sediments (e.g. subglacial stream deposits) Drake (1974), Mills (1977), Benn & Evans (1996), Evans *et al*. (2006), Hart *et al*. (2011)	Disordered clast fabric, coarsest blocks typically along and near the top (‘carapax’); extremely poorly sorted from submicron particles to blocks, clast to matrix‐supported, lenses with larger clasts; local patches with more‐or‐less distinct ‘downstream’ or ‘upstream’ clast imbrication may be present Dufresne *et al*. (2015, 2016), Sanders *et al*. (2018b), Wang *et al*. (2018, 2019)
Clast inventory	Clasts derive from the source area of the debris‐flow deposit; clast spectrum may range from monomictic to polymictic	Depending on size of a glacier or icestream, clasts may be derived from areas >100 km away and/or from a local catchment	Clasts derived from the detachment scar may strongly prevail; clasts derived from other sources (even if distant, e.g. in the case of till) may also be present due to entrainment during transport Hungr & Evans (2004), Dufresne *et al*. (2015)
Clast shape	Clast shape may range from angular to well‐rounded, and any mixtures thereof; shape of clasts is inherited from the source area	Typically a mixture of: (i) faceted clasts with striae; with (ii) angular to subangular clasts produced by subglacial breakage; and (iii) resistant clasts (e.g. eclogite) that acquired their shape from previous processes (e.g. fluvial transport). Relative amount of faceted/striated clasts and broken‐angular and rounded clasts highly variable Hiemstra & Meer (1997), Evans *et al*. (2006), cf. Hart *et al*. (2011)	Angular to subangular pristine rock avalanche material derived from detachment scar; clasts from entrained older sediments may range from angular to well‐rounded Abele (1974), Dufresne *et al*. (2016), Sanders *et al*. (2018b), and many others
Clasts fractured while embedded in deposit	No clasts fractured upon transport	Clasts fractured by glacial overburden locally present. Fractures typically are single or a few, emanate from vertical point contacts between clasts, more rarely from tangential grain loading in simple shear Drake (1972), Hooke & Iverson (1995), Hiemstra & Meer (1997), Hart *et al*. (2011)	Many clasts up to slab size are criss‐crossed by densely‐spaced fractures; in larger clasts, despite dense fracturation, the internal stratigraphy and deformation structures (e.g. folds) are preserved and identifiable. Smaller clasts up to block size display densely fragmented crackle to mosaic fabric; fractures most typically densely criss‐cross clasts; margins of crackle to mosaic clasts may be gradual into their surroundings. Isolated block or blocks throughout riddled by densely‐spaced, single‐cycle macro/microfractures, no distinct vertical and lateral gradient of fracture density McSaveney & Davies (2002), Davies & McSaveney (2009), Anders *et al*. (2013), Dufresne *et al*. (2016), Wang *et al*., 2018), Zeng *et al*. (2019)
Matrix	Depending on source area of a debris flow; may range from mineralogically identical to a prevalent clast fraction in the debris flow to polymictic mineralogy derived from several sources (rocks, older sediments); matrix may be rich in humic material from soil and in organic debris	Except for enclosed rafts of other deposits, basal tills typically are matrix‐supported; matrix results from fracture, attrition, crushing and grinding of clasts, as well as chemical changes in the subglacial setting Fairchild *et al*. (1993), Hiemstra & Meer (1997), Killawee *et al*. (1998),	Matrix content ranges from scarce (= clast‐support) to matrix‐support. Scarce matrices typically consist of silt to sand‐sized, angular rock fragments. Fine‐grained matrices similar to fault gouge are rich in submicron grains. Matrix is mineralogically identical, or nearly so, to the transported pristine rock‐avalanche material Dufresne *et al*. (2015, 2016), Davies *et al*. (2020)
Cataclastic shear belts	Absent	Absent. Glaciotectonic features produced by basal shear may appear broadly similar to shear‐induced features along the base of rock avalanches propagating over soft deformable substrate Dreimanis (1993), Benn & Evans (1996), Evans *et al*. (2006), Aber & Ber (2007), Hart *et al*. (2011)	Planar features up to *ca* 30 cm in width with a matrix of ultracataclastic gouge and small clasts typically up to fine pebble size. Fabric ranges from clast to matrix‐supported. Shear belts are typically folded or faulted and comprise strongly elongate lenses; may contain a few clasts that are polished and striated by the process of rock avalanching Dufresne *et al*. (2016), Zeng *et al*. (2019)

**Fig. 12:**
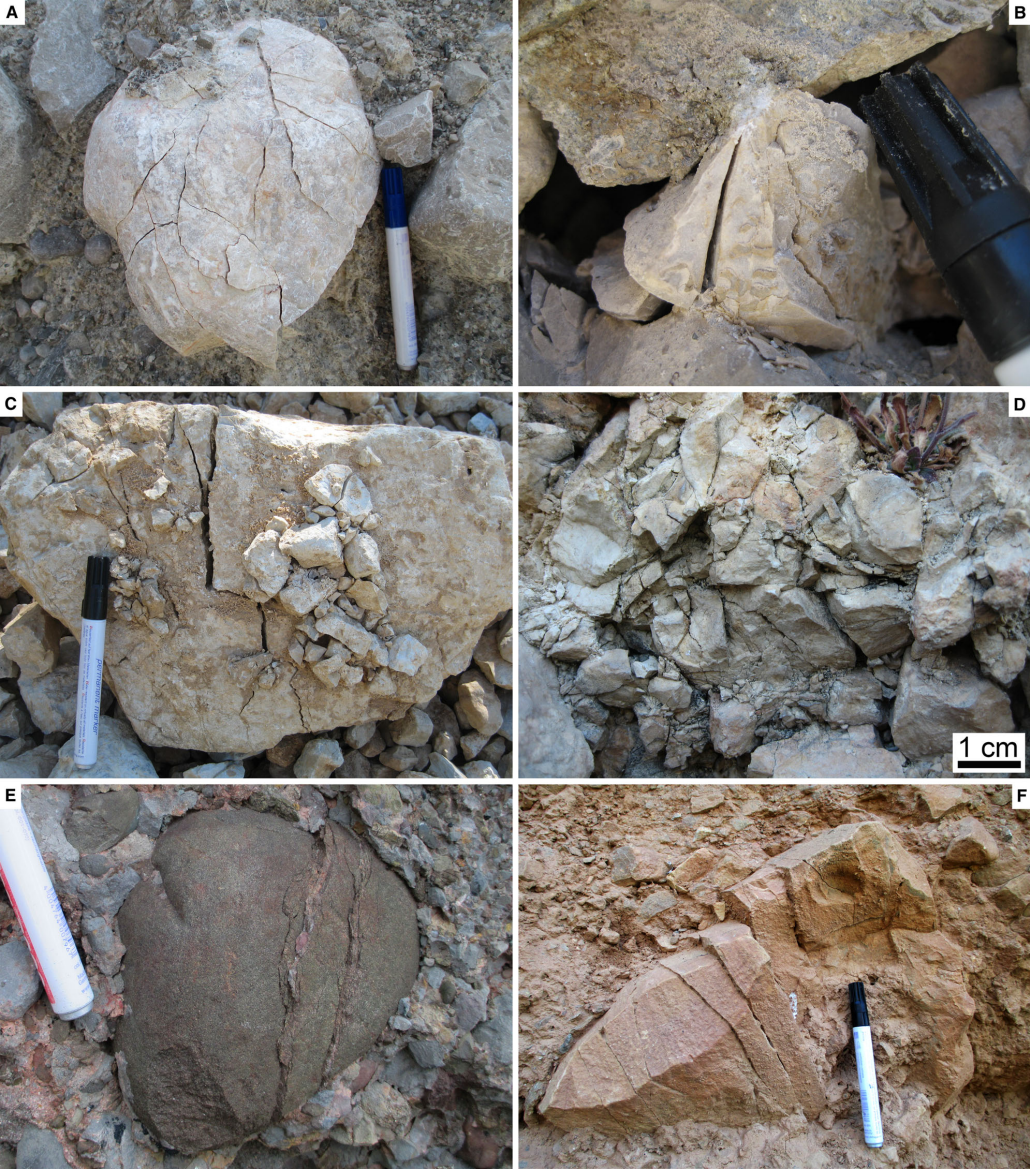
Comparative images of clasts fractured in contexts unrelated to rock avalanching (see Data S5 for additional information). (A) Till with a cobble cracked by glacial loading. Pen: 14 cm. Hinterriss, Eastern Alps. (B) Pebble fractured at vertical point contacts due to coseismic acceleration. Urschenbach talus fan, Eastern Alps. Pen tip 14 mm wide. (C) Cobble cracked by deformation close to a normal fault. Lithified talus along Assergi fault, Apennines. (D) Pebble crushed to a crackle fabric near a normal fault. Lithified talus at Camarda, Apennines. (E) and (F) Clasts fractured and sheared in syntectonic molasse conglomerates. Sant Llorenç de Moryuns (E) and Riglos (F), southern Pyrenees.

Clasts fractured *in situ* are also known from subglacial till and proglacial pebbly deposits subject to glacial overburden. Aside from the overall distinct characteristics of proglacial–fluvial and subglacial sediments, broken clasts in these deposits typically display a single or a few fractures that are oriented subvertically (in some cases in conjugate sets), and are associated with vertical point contacts between clasts (Fig. 12A). Another process of fracturation of clasts while embedded in sediment is coseismic deformation. Again, also in these cases, fractures typically are single or a few, are oriented subvertically, and typically emanate from vertical point contacts (Fig. 12B and C) (Costantini & Ortner, 2013; Sanders, 2016). Coseismic cracks, typically as single fractures, in many cases extend subvertically over *several* clasts that are in point contact (Meyer *et al*., 2006). Close to faults, complete crushing of smaller clasts resulting in a crackle to jigsaw fabric is locally observed. In this case, the sediment in which the crushed clast is embedded may be clearly not of rock‐avalanche origin (Fig. 12D). Finally, fracturation in syntectonic strata typically is confined to larger clasts. Fractures are relatively straight and associated with a ‘systematic’ offset of fracture fragments (Fig. 12E and F) (cf. Eidelman & Reches, 1992; Tokarski & Strzelecki, 2020). In contrast to the discussed mechanisms of *in situ* clast fracturation, crackle to mosaic clasts in CMWs typically are criss‐crossed by numerous fractures and may display a more‐or‐less distinctly ‘exploded’ appearance due to slight translation/rotation of subclast fragments relative to one another (Figs 4, 7A to 7E, 11B and 11C). To sum up, in particular if they are common, crackle to jigsaw to mosaic clast fabrics such as those observed in the RB are perhaps the most diagnostic feature of a rock‐avalanche origin. Crackle to mosaic clast fabrics result from dynamic disintegration by mutual clast collisions during rock‐avalanche propagation. Evidence for dynamic disintegration of clasts, thus, reflects the much higher kinetic energy that distinguishes rock‐avalanche propagation from other types of mass flows, such as debris flows (see, e.g., Dufresne *et al*., 2016; Antonielli *et al*., 2020). Clasts that became fractured while embedded in a deposit (= fractured *in situ*) also are known from coseismic deformation or from shearing under glacial load in basal tills. Coseismic clast fracture, however, tends to produce subvertical open fracture networks confined to the vicinity of fault zones (Meyer *et al*., 2006), or a pattern of clast comminution leading to a densely packed, partially modified sediment fabric (Sanders *et al*., 2018). Cracking of clasts by subglacial loading/shearing in proglacial gravels that were overridden by ice streams most typically is confined to a few cracks – associated with little or no translation of fracture fragments – across specific clasts that either were of a relatively ‘weak’ lithology (for example, limestone among clasts of competent metamorphites, such as eclogites) or an orientation of the clast relative to overburden that favoured cracking, such as a mica schist in point contact to more competent clasts. In basal tills, in turn, clasts fractured *in situ* typically are confined to a few clasts in point contact with one another **(**Hiemstra & Meer, 1997; Hart *et al*., 2011). In addition, except for the carapace of rock avalanches wherein clasts had been sufficiently free to move to prevent fracturation by mutual collision, it is mainly the pervasiveness, or abundance, of clasts fractured *in situ* that distinguishes rock‐avalanche deposits from other sediment types (see below for further discussion).

From young RADs, steeply inclined clast [a,b]‐planes and imbricate fabrics are documented. Along a RAD, however, the facing of tilting and of imbricate structures is not necessarily constantly downstream or upstream, but may change (Wang *et al*., 2020). Thus, the steep‐tilted clasts in the RB do not indicate a specific transport direction (cf. Fig. 6C and E). The described quasi‐strata in the RB that are deformed and folded (Figs 7F, 8A and 8B) are interpreted as ‘shear belts’ as observed in well‐exposed young rock‐avalanches (Dufresne *et al*., 2016; Zeng *et al*., 2019).

### Clast inventory

A comparison of the clast inventory of the RB with substrate geology strongly suggests that the rock mass detached from the northern basin margin, i.e. the front range. Along the northern margin, in the area between Monte Tremoggia and Monte Prena: (i) Upper Triassic dolostones (Dolomia Principale, Dolomie Bituminose); and (ii) Jurassic limestones of deep‐water environments are exposed (Figs 1B, 1C and 2) (see Vezzani & Ghisetti, 1998; Section 4 in Ghisetti & Vezzani, 1986; Section C in fig. 3 of Satolli *et al*., 2005). Although the precise geomorphological configuration of the front range at the time of rock avalanching is not known, it can be assumed that upon detachment of a rock avalanche, these lithologies should have been available for downslope transport.

Conversely, a detailed field check and inspection of six samples in thin section from the southern flank of the Campo Imperatore basin between Cima di Monte Bolza and Monte Bolza (cf. Fig. 1C; Fig. 2) revealed no obvious fit of microfacies with the lithologies observed in the RAD. Along the mentioned southern basin flank, chiefly shallow‐water bioclastic grainstones to rudstones, bioclastic packstones and, subordinately, bioclastic wackestones are exposed (Formazione di Terratta and Calcare Massiccio, see Table 1, Fig. 2). Moreover, the southern basin flank is onlapped by a suite of highly fossiliferous Miocene–Pliocene neritic deposits (Fig. 2, Table 1). No clasts of these well‐identifiable rocks have been found in the RB. This underscores a northerly derivation of the rock avalanche, perhaps from the area of the present Fornaca catchment (Fig. 1C).

As described, the clast spectrum of the Old Conglomerate is dominated by dolostones (see Data S2). At least in the present configuration of the Campo Imperatore basin, dolostone clasts can be derived from the Upper Triassic Dolomia Principale and Dolomie Bituminose units exposed in the Fornaca catchment in the front range. In addition, dolomitized Lower Jurassic Calcare Massiccio exposed in the front range of the north‐westernmost part of the basin west of Monte Brancastello (dolomitized portions of this formation are not shown in Fig. 2) also delivers dolostone clasts to alluvial fans, but it is not expected that the dolostones of the north‐westernmost part of the basin contributed to the clast inventory of the studied RB. Calcare Massiccio is also exposed along a sector of the southern basin margin along Valle Cortina (Fig. 2, Table 1) where, however, this unit consists of limestone. As described, at Colle Caciaro, the Old Conglomerate pinches out in onlap onto a distinct relief of the Calcare Massiccio bedrock, and the RB directly overlies the latter (Fig. 3). This suggests that at least a part of the clasts of shallow‐water bioclastic limestones in the RB may have been entrained during rock avalanche descent. A derivation of at least a major share of the clastic sediment of the RB from the front range would also fit with the fairly common presence of clasts of proto‐/cataclasites. The more diversified clast spectrum of the Old Conglomerate relative to that of the Rubble Breccia strongly suggests that the conglomerate was supplied not only from the Fornaca catchment, but includes clasts derived from much or all of the terrain upstream of the study area, and that may include clastic sediment from both the northern and southern basin margins (cf. Fig. 2). As mentioned, the presently active Fornaca alluvial fan is strongly dominated by dolostone clasts derived from erosion of, both, the Dolomia Principale and the Dolomie Bituminose unit as exposed in the majority of the present Fornaca catchment.

Where rock avalanches propagate over deformable substrate, such as alluvial pebbles and sands or lacustrine strata, the substrate is most commonly deformed by folding and shearing and/or comprises diapir‐like structures (Erismann & Abele, 2001; Dufresne *et al*., 2016). In addition, more‐or‐less large rafts of foreign material or disseminated substrate material may be entrained into rock avalanches (Dufresne *et al*., 2016). Along the contact of the Old Conglomerate to the overlying RB (cf. Fig. 5), in contrast, both in field and thin section, no features of ductile deformation of the conglomerate related to rock avalanching have been observed. Similarly, no evidence for entrainment at least of significant amounts of older clastic material and other features observed in rock avalanches that run across soft substrates (such as injection dykes; see, e.g. Dufresne *et al*., 2016) were seen. Rock avalanches that propagate over deformable substrates (for example, alluvial deposits) may entrain a highly variable amount of the overridden material into the moving mass. Relative percentages of entrained versus original rock material range from nearly zero to several hundreds of percent of the originally detached rock mass that gave rise to catastrophic mass‐wasting (e.g. Hungr & Evans, 2004; Zeng *et al*., 2019). Clasts entrained during rock‐avalanche propagation may be identified by a shape suggestive of other depositional processes that preceded rock‐avalanching (for example, rounded pebbles cut by fractures) and/or by a lithology that does not fit with lithologies from the detachment scar of a rock avalanche (Sanders *et al*., 2021). In case of a palaeo‐rock avalanche as described herein, however, the detachment scar may have been significantly overprinted by erosion, such that it is not straightforward to assign lithologies found within a fossil RAD to the lithological composition in the area of the assumed detachment scar. In some cases, entrained material is easy to identify by a lithological composition and fabric strongly different from the rest of the RAD, such as podiform bodies of polymictic, alluvial siliciclastic material entrained into an overall carbonate‐lithic RAD (Patzelt, 2012b; Dufresne *et al*., 2016). In our case, no large enclosures of obviously foreign material were identified. Instead, the described comparison of the lithologies exposed along the northern and southern basin margin suggest that the rock avalanche detached from the northern margin, and mainly consists of lithologies comparable with the front range.

An additional way to test for material entrainment is a comparison of the lithologies of a RAD and the lithologies in the overridden succession. A strictly quantitative analysis of the clast lithologies in the Old Conglomerate that underlies the RAD was not undertaken here. Evidence from thin sections of the Old Conglomerate, however, indicates a much more diverse clast spectrum than was observed in slabs and thin sections of the overlying RAD. As described, the Old Conglomerate most probably was lithified when overridden by the rock avalanche. Together, this evidence strongly suggests that the Old Conglomerate contributed little material, if any, to the RAD.

As mentioned, no evidence was found for soft‐sediment deformation of the Old Conglomerate near the contact to the RB, but the contact clearly is unconformable relative to the subhorizontal bedding in the conglomerate. This unconformable contact may have been produced by subaerial erosion of the Old Conglomerate well before rock avalanching, or by dynamic erosion and material entrainment from the rock avalanche. Where the unconformity is well‐exposed and accessible, such as on Colle Caciaro, the marked difference in degree of cementation between the top of the Old Conglomerate and the overlying Rubble Breccia scarce in cement suggests that the conglomerate was lithified before rock‐avalanche descent. This may help to explain why the rock avalanche seems to have entrained little foreign material, at least in the exposed sector. As described, the top of the Old Conglomerate displays relicts of a thick fringe of radial‐fibrous calcite spar that are engulfed by and overlain by poikilotopic spar (Fig. 5), indicating that the fibrous cement became replaced by the latter. It is not clear, however, whether all of the poikilotopic spar originated by replacement of a precursor cement. Poikilotopic spar records a phreatic diagenetic environment (e.g. Moore, 1989; James & Jones, 2016), yet burial required for precipitation does not necessarily need to be deep as long as phreatic conditions and sufficient pore‐water flow prevail; poikilotopic spar thus is also a characteristic feature of groundwater calcretes formed only a few metres below the surface (e.g. Nash & Smith, 1998; Stoops *et al*., 2010).

### Diagenesis and age

Subaerial CMWs that consist of carbonate rocks or of rocks with carbonate minerals, such as many types of metamorphics, may undergo partial lithification very early after emplacement (Ostermann *et al*., 2007, 2017; Sanders *et al*., 2010). Because catastrophic mass‐wasting results in production of abundant nanometre to micrometre‐scale rock particles (Sanders *et al*., 2010; Davies *et al*., 2020), the huge reactive surface provided by these results in rapid dissolution upon influx of meteoric waters, and reprecipitation as diverse forms of diagenetic carbonates ranging from microspar to small speleothems. The presence of cements provides the possibility to obtain an *ante‐quam* age of the mass‐wasting event with the U/Th disequilibrium method (Ostermann *et al*., 2007, 2017); this holds in particular for partially eroded fossil RAD that can no longer be dated with ^36^Cl exposure dating of boulders or with radiocarbon dating of enclosed organic remnants (Sanders *et al*., 2010).

The two main diagenetic fabrics in the RB, i.e.: (i) secondary dissolution porosity; and (ii) pendant cements and isopachous cements were produced in a meteoric diagenetic environment. Small relicts of ultracataclastic matrix within the interstitial space (Figs 8E, 8F, 9A and 9B) suggest that the primary fracture pore space originally contained more of, or was filled by, this matrix that, however, was dissolved upon percolation of meteoric waters. Further indications of dissolution are provided by lithomouldic pores (Fig. 9B to D) and by small relicts of cataclastic matrix floating within cement (Fig. 10B). As mentioned, cement is generally scarce in the RB and typically confined to small pendant fabrics (Fig. 9D to F). This suggests that, after partial early dissolution mainly of scarce matrix, the entire diagenetic system was relatively open such that the RB was quite permeable to meteoric‐derived waters. The relicts of the Perched Fan deposits above the RB (cf. Fig. 3) suggest that, before relative uplift on the fault‐bounded horst, the latter was buried under alluvial sediments. Due to erosion of the top of the RB and of the Perched Fan deposit, a good quantitative estimate of former burial is not possible. It is suggested here that the rock avalanche deposit underwent a diagenetic cycle including: (i) Subaerial rock‐avalanche deposition followed by dissolution mainly of fine‐grained cataclastic matrix in a meteoric‐vadose environment, accompanied by precipitation of pendant cements. (ii) Upon further subsidence due to downthrow along the master fault system of the Campo Imperatore basin, the RAD became buried by alluvial‐fan deposits. Both, fault‐induced subsidence and burial by alluvial‐fan deposits, led to phreatic diagenetic conditions within the RAD, as recorded by isopachous fringes of cement on clasts and by pore‐fillings of calcite orthospar. Locally and/or subsequently, meteoric dissolution of older cements changed with precipitation of younger cements. (iii) Finally, relative uplift across the Cucchiarelle normal fault (cf. Fig. 3) led to a return of vadose conditions as recorded by dissolution of matrix, clasts and all of the older cements. Inasmuch this cycle was also the result of wet/dry climatic changes cannot be assessed with the data at hand.

The calcite cement that could be sampled for U/Th dating (Fig. 10A and B) post‐dates an earlier cement generation that, in turn, underwent a period of dissolution before renewed precipitation took place (Fig. 10C and D). Furthermore, the observation that the dated cement fills fractures in the RB (Fig. 10A) indicates that this cement precipitated when the breccia was sufficiently lithified to deform in a brittle fashion, perhaps during an earthquake related to faulting (cf. Fig. 3).

**Fig. 13:**
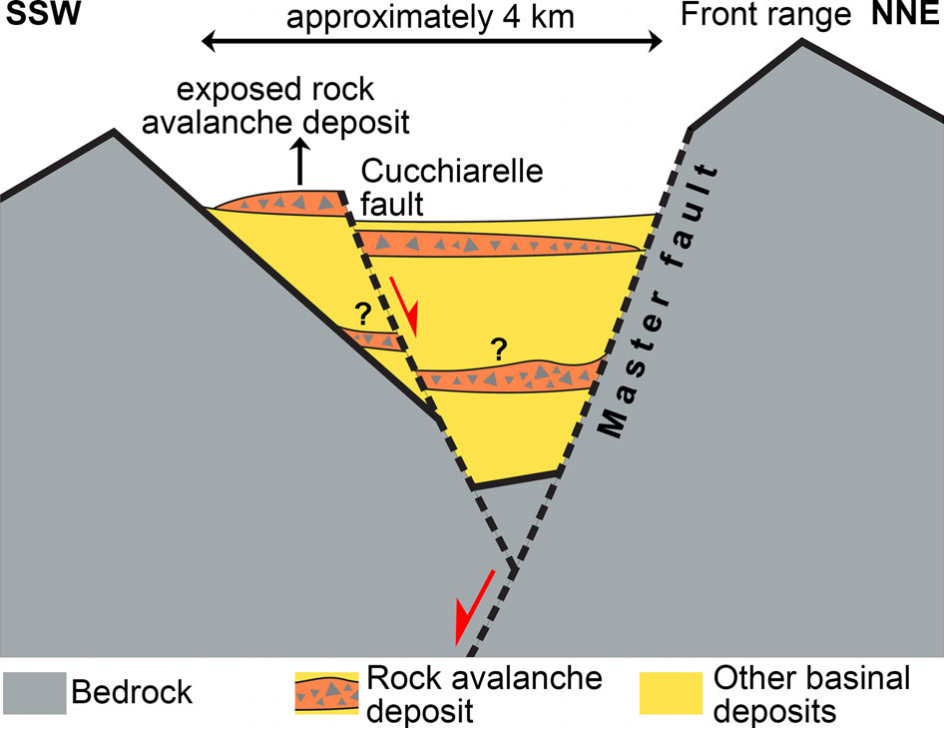
Schematic cross‐section through the Campo Imperatore basin (cf. Calembert *et al*., 1972) as a representative of similar basins in general. The fossil rock‐avalanche deposit described herein became exposed by activity of the antithetic Cucchiarelle fault system. It is speculated that rock avalanche deposits are widespread in the fillings of intramontane basins.

Today, the RB and the overlying Perched Fan deposits are exposed on a horst bounded by the Cucchiarelle fault system; the drainage of the Fornaca alluvial fan bypasses the horst at fault oversteps (Fig. 3). As mentioned, there is no evidence for significant clastic input from the southern basin margin. Before faulting, as obvious from the Perched Fan deposits, the northerly‐sourced Fornaca fan had reached the southern basin margin. The calcite spar used for U/Th dating formed in a phreatic diagenetic setting and later became exposed on the fault horst produced by the activity of the Cucchiarelle faults. It cannot be stated whether the Cucchiarelle faults existed before, but the U/Th age of the cement suggests that these faults came into existence, or increased in activity, at least since *ca* 124 ka. Until today, the accommodation space produced by faulting is not compensated by alluvial fan aggradation. Under the assumption that the rock avalanche of the studied deposit detached from an area roughly corresponding to the position of the present Fornaca catchment along the northern front range (cf. Fig. 1C), the runout amounts to approximately 4 km or slightly more (Fig. 13). Because the volume of the original rock avalanche deposit is not known, and because erosion since at least 124 ka (see Data S3) had wiped out any clear‐cut shape of a detachment scar, no further inferences on the relation between volume and runout of the old rock avalanche can be made.

### Concluding notes

With respect to the geological preservation of subaerial CMW deposits, it is common practice to think that, because they are present in mountain ranges, their preservation potential is near to zero. Whereas this may hold for mountain ranges undergoing rapid prolonged uplift such as the Southern Alps of New Zealand (but see McColl, 2020), this does not apply to ranges subject to localized fault‐related subsidence such as the European Eastern Alps or the Apennines.

In a study of eight large gravitational displacements (mainly slow deep‐seated gravitational slope deformations) in the central Apennines, Galadini (2006) identified normal faults as a major cause of slope instability, in that these: (i) produce local relief; and (ii) favour large‐scale mass wasting by seismic activity or merely by providing sliding planes. In a study of young rock avalanches in the central Apennines, Bianchi Fasani *et al*. (2014) identified two main structural settings favourable for rock‐avalanche detachment, i.e.: (i) rock avalanches detached from the forelimbs of frontal anticlines; and (ii) rock avalanches that detached from the backlimbs of anticlines cut by normal faults of large throw, or from older thrust planes reactivated as normal faults. The rock avalanche reconstructed herein would pertain to the latter structural setting, i.e. catastrophic mass‐wasting detached from the backlimb of an anticline cut by normal faults. In a reflection seismic section across part of the L'Aquila–Scoppito subbasin (of the L'Aquila basin) *ca* 15 km west of the Campo Imperatore basin, Cosentino *et al*. (2017, figs 5 and 12) indicate a lens‐shaped subsurface unit labelled as ‘chaotic breccias and megabreccias'. At least along the seismic trace, this unit does not onlap a normal fault, but occupies an isolated position within the basinal stratigraphic succession. These features are compatible with an origin from catastrophic mass‐wasting (see also Cosentino *et al*., 2017). The examples from the Scoppito basin and from the Campo Imperatore basin suggest that ancient CMW deposits may be relatively common in the subsurface of intramontane basinal successions (Fig. 13). In the L'Aquila basin close to the study area, Antonielli *et al*. (2020) identified a widespread, blocky carbonate‐lithic deposit (L'Aquila Breccia) up to 130 m in thickness as a fossil rock avalanche that detached from the former southern slope of the Gran Sasso range, probably in relation to the Assergi normal fault system. In contrast to the RAD described herein, the L'Aquila Breccia is exposed at surface over some 13.5 km (see Antonielli *et al*., 2020, fig. 3) which, despite post‐depositional erosion, may represent at least the largest part of its original extent. Also in case of the L'Aquila Breccia, the interpretation of the deposit was uncertain until the validation of the hallmark feature of clasts fractured *in situ* led to the new interpretation in terms of a rock avalanche deposit (Antonielli *et al*., 2020). Provided that the L'Aquila Breccia indeed resulted from a single catastrophic mass‐wasting, this event had an unusually long runout of at least 13.5 km (cf. Antonielli *et al*., 2020, fig. 3), but similar runouts of up to 15 km are known from post‐glacial rock avalanches in the Alps (for example, the channelized medial to distal part of the Tamins rock avalanche, see Data S5). Based on numerical ages in underlying and overlying deposits, the rock avalanche that gave rise to the L'Aquila Breccia is age‐bracketed between 365 ka and 126 ka (Antonielli *et al*., 2020).

Fossil CMW deposits may be easily overlooked because they are erosionally truncated and draped by younger sediments, or because they are preserved only as erosional vestiges at the surface, or because they are mistaken for other sediments, in particular for debris flows (cf. Schultz, 1986; Yarnold, 1993; Gruber *et al*., 2009). In mountain ranges, if accommodation space is produced by faulting, local base‐level rise should markedly enhance the preservation potential of fossil CMW. To date, fossil CMW deposits are age‐bracketed by overlying and/or underlying sediments (Yarnold, 1993; Antonielli *et al*., 2020). In many cases, however, not even this is possible. Aside from its overall scarcity in cement and difficulties with a complex post‐depositional diagenetic history, the RB thus highlights the potential of the U/Th method to deduce age constraints from CMW deposits that are unsuited for any other dating method. Identification of fossil CMWs means not mere reinterpretation of a lithosome, but bears implications with respect to palaeorelief, to processes of erosion and deposition and, in case supportive other records (for example, lakes, speleothems) are available, to potential palaeoseismicity.

## CONCLUSIONS

1. In the Campo Imperatore halfgraben basin of the central Apennines, an interval of breccia previously assigned, once, to glacial moraines and, at another time, to alluvial fan sediments has been reinterpreted as a deposit of a ‘fossil’ (pre‐last glacial) rock avalanche. The rock avalanche deposit – dubbed Rubble Breccia – overlies a succession of Pleistocene alluvial conglomerates and, in turn, is erosionally truncated and locally overlain by unlithified alluvial fan deposits perched above a presently‐active alluvial fan due to normal faulting.
2. The Rubble Breccia throughout is characterized by: (i) an extremely‐poorly sorted, disordered fabric of angular clasts of silt to boulder size; in addition, (ii) pebbles to boulders that became densely fragmented while embedded within the sediment, resulting in crackle, jigsaw and mosaic fabrics, respectively, are common; and finally (iii) planar features weathering proud of the surface, interpreted as transiently active shear belts within the moving rock avalanche, that are deformed into open to recumbent to convolute folds, respectively, are present. The clasts of the Rubble Breccia fit with lithologies exposed along the northern basin flank.
3. In the local geological context, a U/Th disequilibrium age of 124.25 ± 2.76 ka bp of a fracture‐filling cement in the Rubble Breccia suggests that the normal fault that led to relative uplift and exposure of the entire Pleistocene succession (alluvial conglomerates to unlithified fan deposits) came into existence, or increased in activity, *at least* since *ca* 124 ka.
4. For fossil rock avalanche deposits no longer evident by surface morphology due to partial erosion or burial by younger deposits, the following features are considered diagnostic: (i) common presence of crackle, jigsaw and mosaic fabrics mainly after precursor cobbles to blocks, embedded in: (ii) an extremely‐poorly sorted groundmass of disordered, most commonly angular clasts of sand to cobble to boulder size, and with an ultracataclastic matrix; the groundmass may show: (iii) elongate shear belts that may contain more fine‐grained cataclastic matrix than their surroundings, and that may be deformed into open folds to convolute folds. Due to potential entrainment of foreign material during runout, its presence is not necessarily an argument against a rock avalanche, provided that the criteria listed above are fulfilled.
5. Catastrophic mass‐wasting (CMW) requires sufficient relief amplitude and competent rocks to form. Mountain ranges are not only areas of uplift and erosion, but may undergo significant extensional and/or strike‐slip faulting resulting in intramontane basins, enhancing long‐term preservation. Fossil CMW deposits should be more common in the rock record than is acknowledged to date, but perhaps were mistaken for other rocks or sediments, such as cataclasites, tills and debris flows.

## CONFLICT OF INTEREST

We declare that we do not have any commercial or associative interest that represents a conflict of interest in connection with the work submitted.

## Supporting information


**Data S1**. Table listing the clast types of samples composed mainly of limestones from the Rubble Breccia.

**Data S2**. Table listing the clast types identified in the ‘Old Conglomerate’ underlying the Rubble Breccia.

**Data S3**. Table showing the data behind the U/Th age determination of a calcite cement, the final calculated ages, and the references the age calculation was based on.

**Data S4**. ‘Rosholt diagrams’ to the U/Th age of the dated calcite cement.

**Data S5**. Table showing background data and references to the interpretation of the deposits discussed in the text.

## Data Availability

The data that support the findings of this study are available from the corresponding author upon reasonable request.
