# Supplementary material for: Identification of a Quaternary rock avalanche deposit (Central Apennines, Italy): Significance for recognition of fossil catastrophic mass‐wasting
Source: Sedimentology. 2022 Mar 29;69(5):2099–130. doi: 10.1111/sed.12984 (PMC9541593; doi:10.1111/sed.12984)
Supplement: Supplementary file 1 — Data S1. Table listing the clast types of samples composed mainly of limestones from the Rubble Breccia. [file SED-69-2099-s003.docx]

**Table S1**. Clast types of limestone samples from the Rubble Breccia.

| **Sample code** | **Clast types** | **Interpretation** |
| --- | --- | --- |
|  |  |  |
| **CI 46** | Entire thin section of:  Peloidal grst with smaller benthic foraminifera (Textularina, Miliolina). | Shallow-water platform  (Jurassic-Cretaceous) |
|  |  |  |
| **CI 47** | Entire thin section of:  Fine-grained bioclastic wkst with tiny echinoderm ossicules (presumably of *Saccocoma*), smaller benthic foraminifera (Rotalina), radiolarians and a few disarticulated ostracod tests | Deep water depositional environment  (Middle-Upper Jurassic?) |
|  |  |  |
| **CI 50** | Entire thin section of:  Peloidal grst with a few smaller benthic foraminifera, dasycladalean alga, and aggregate grains | Shallow-water platform  (Jurassic-Cretaceous) |
|  |  |  |
| **CI 60A** | Entire thin section of:  Micropeloidal pkst with radiolarians and tiny echinoderm ossicules (presumably of *Saccocoma*) | Deep water depositional environment (Upper Jurassic?) |
| **CI 60B** | (a) Micropeloidal pksts with radiolarians and tiny echinoderm ossicules (presumably of *Saccocoma*)  (b) Sparry ultracataclasite | (a) Deep water depositional environment (Upper Jurassic?)  (b) Fault rock |
| **CI 60C** | (a) Micropeloidal pksts with radiolarians and tiny echinoderm ossicules (presumably of *Saccocoma*)  (b) Micropeloidal pksts-wkst with sponge spicules  (c) Mixed peloidal/echinoderm-clastic pkst-grst | (a) Deep water depositional environment (Middle-Upper Jurassic?)  (b) Deep water depositional environment  (Jurassic?)  (c) Deep water depositional environment  (Jurassic, ?Cretaceous) |
|  |  |  |
| **VCO 3** | (a) Mixed peloidal/shallow-water bioclastic grsts, some with *Tubiphytes* fragments  (b) cataclasites  (c) dolostone clasts | (a) Shallow-water platform (probably Jurassic)  (b) Fault rocks  (c) Dolostones, presumably after platform limestones |
|  |  |  |
| **VCO 4** | Entire thin section of:  Mixed peloidal/shallow-water bioclastic grst | Shallow-water platform (Jurassic-Cretaceous) or calciturbidite |
|  |  |  |
| **VCO 6** | Entire thin section of:  Bioclastic pkst-wkst with small echinoderm fragments, smaller benthic foraminifera (Rotalina), radiolarians, and a few disarticulated ostracod tests | Deep water depositional environment (Jurassic-Cretaceous) |
|  |  |  |
| **VCO 11** | (a) Mixed bioclastic/peloidal grsts with benthic foraminifera (Textularina, Miliolina)  (b) a few clasts of dolostone | (a) Shallow-water platform (Jurassic-Cretaceous?) or calciturbidite  (b) Dolostones, presumably after platform limestones |
|  |  |  |
| **VCO 30** | (a) Cataclasites and (sparry) ultracataclasites  (b) Bioclastic pksts | (a) Fault rocks  (b) Calciturbidite |
