# Supplementary material for: Identification of a Quaternary rock avalanche deposit (Central Apennines, Italy): Significance for recognition of fossil catastrophic mass‐wasting
Source: Sedimentology. 2022 Mar 29;69(5):2099–130. doi: 10.1111/sed.12984 (PMC9541593; doi:10.1111/sed.12984)
Supplement: Supplementary file 2 — Data S2. Table listing the clast types identified in the ‘Old Conglomerate’ underlying the Rubble Breccia. [file SED-69-2099-s002.docx]

**Table S2**. Clast types in the Old Fan Conglomerate (cf. Fig. 3, Table 2). The clasts are listed per facies type, not by their relative amount or number, except for the obvious prevalence of dolostone clasts in all samples.

| **Sample code** | **Clast types** |
| --- | --- |
|  |  |
| **VC 3** | Medium-grained to coarse-sparry dolostones (main sediment fraction)  Partly dolomitized peloidal grainstone  Bioclastic wackestone with benthic foraminifera  Lime mudstone with globotruncanids  Dolocataclasite |
|  |  |
| **VC 5** | Medium-grained to coarse-sparry dolostones (main sediment fraction)  Boundstone with Tubiphytes  Rudist-clastic packstone  Radiolarian wackestone  Shallow-water bioclastic grainstone  Wackestone with calcispheres and globotruncanid foraminifera  Wackestone with Lower Cretaceous planktic foraminifera (?*Rotalipora*)  Wackestone with globorotalid foraminifera  Isolated fragment of radiolitid rudist  Chert |
|  |  |
| **VC 21** | Medium-grained to coarse-sparry dolostones (main sediment fraction)  Cementstone with shallow-water bioclastic material  Shallow-water bioclastic packstone  Lime mudstone  Isolated fragment of radiolitid rudist  Shallow-water bioclastic grainstone  Rudist-clastic packstone  Peloidal grainstone  Wackestone with globotruncanid foraminifera  Chert |
|  |  |
| **VC 31** | Medium-grained to coarse-sparry dolostones (main sediment fraction)  Dolo-Protocataclasite  Fault cataclasite (limestone)  Lithic packstone (fragment of calciturbidite?)  Wackestones with globorotalid foraminifera  Wackestones with globotruncanid foraminifera  Packstone with globorotalid foraminifera  Lime mudstone with small ?ostracods  Shallow-water bioclastic grainstone with benthic foraminifera  Oolithic grainstone  Chert |
|  |  |
| **VCO 1** | Medium-grained to coarse-sparry dolostones (main sediment fraction)  Coral boundstone  Shallow-water bioclastic grainstone  Peloidal-bioclastic packstone with echinoderm fragments  Spiculithic wackestone  Dolomitized ?coral  Wackestone with globorotalids  Peloidal-bioclastic grainstone  Peloidal grainstone  Chert |
|  |  |
| **VCO 24** | Medium-grained to coarse-sparry dolostones (main sediment fraction)  Shallow-water bioclastic grainstones  Rudist-clastic wackestones  Peloidal grainstone  Mixed oolithic-bioclastic grainstone  Calpionellid mudstone  Calpionellid wackestone |
|  |  |
| **VCO 32** | Medium-grained to coarse-sparry dolostones (main sediment fraction)  Radiolarian mudstone  Oolithic grainstone  Protocataclasite  Packstone with rudist fragments  Wackestone with globotruncanid foraminifera |
