# Supplementary material for: Identification of a Quaternary rock avalanche deposit (Central Apennines, Italy): Significance for recognition of fossil catastrophic mass‐wasting
Source: Sedimentology. 2022 Mar 29;69(5):2099–130. doi: 10.1111/sed.12984 (PMC9541593; doi:10.1111/sed.12984)
Supplement: Supplementary file 3 — Data S3. Table showing the data behind the U/Th age determination of a calcite cement, the final calculated ages, and the references the age calculation was based on. [file SED-69-2099-s001.docx]

**Table S3**. MC-ICP-MS U-Th dating results of samples analysed in this study.


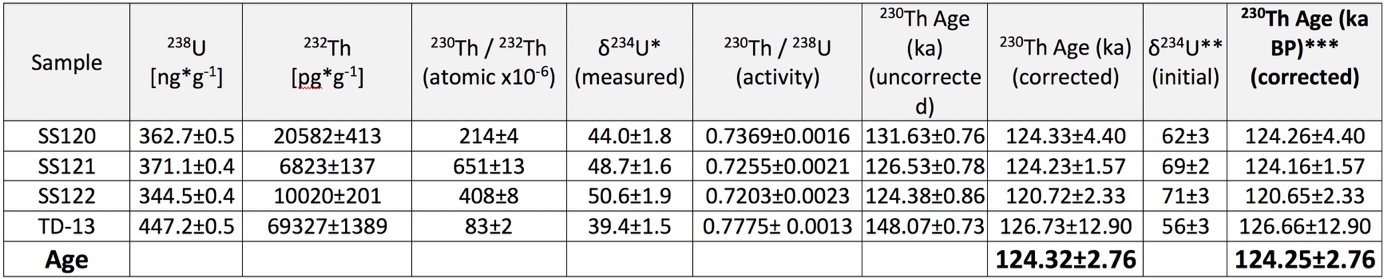


λ_230_ = 9.1705 x 10^-6^ a^-1^ (Cheng et al., 2013)

λ_234_ = 2.8221 x 10^-6^ a^-1^ (Cheng et al., 2013)

λ_238_ = 1.551 x 10^-10^ a^-1^ (Jaffey et al., 1979)

λ_232_ = 4.944 x 10^-11^ a^-1^ (LeRoux and Glendenin, 1963)

* δ^234^U = ([^234^U/^238^U]_activity_ – 1)x1000.

** δ^234^U_initial_ was calculated based on corrected ^230^Th age (T), i.e., δ^234^U_initial_ = δ^234^U_measured_ * e^λ234*T^.

***B.P. stands for ‘Before Present’ where the ‘Present’ is defined as the year 1950 A.D.

The isochrone age was derived following the methodology described in Vermeesch (2018)[ using the maximum likelihood regression of Ludwig and Titterington (1994)]. Corrected ^230^Th ages use the initial ^230^Th/^232^Th atomic ratio of 2.00 ± 0.84 x10^-5^.

**References**

**Cheng, H., Edwards, R.L., Shen, C.-C., Polyak, V.J., Asmerom, Y., Woodhead, J., Wang, Y., Kong, X., Spötl, C., Wang, X.** and **Alexander, E.C.** (2013) Improvements in ^230^Th dating, ^230^Th and ^234^U half-life values, and U-Th isotopic measurements by multi-collector inductively coupled plasma mass spectrometry. *Earth Planet. Sci. Lett.* **371–372**, 82–91.

**Jaffey, A.H. Flynn, K.F., Glendenin, L.E., Bently, W.C.** and **Essling, A.M.** (1979) Precision measurement of half-lives and specific activities of ^235^U and ^238^U. *Phys. Rev. C 4* **1889** (1979).

**Le Roux, L.J.** and **Glendenin, L.** (1963) Half-life of ^232^Th. Proc. Ntl. Mtg. Nuclear Energy, Pretoria, South Africa, **83**, p. 94.

**Ludwig, K.R.** and **Titterington, D.M.** (1994) Calculation of ^230^Th/U isochrons, ages, and errors. *Geochim. Cosmochim. Acta*, **58**, 5031-5042.

**Vermeesch, P.** (2018) IsoplotR: a free and open toolbox for geochronology. *Geoscience Frontiers*, **9**, 1479-1493.
