# Supplementary figures and images for: Identification of a Quaternary rock avalanche deposit (Central Apennines, Italy): Significance for recognition of fossil catastrophic mass‐wasting
Source: Sedimentology. 2022 Mar 29;69(5):2099–130. doi: 10.1111/sed.12984 (PMC9541593; doi:10.1111/sed.12984)

**Data S4**. Rosholt diagrams to the U/Th ages shown in Table S3.


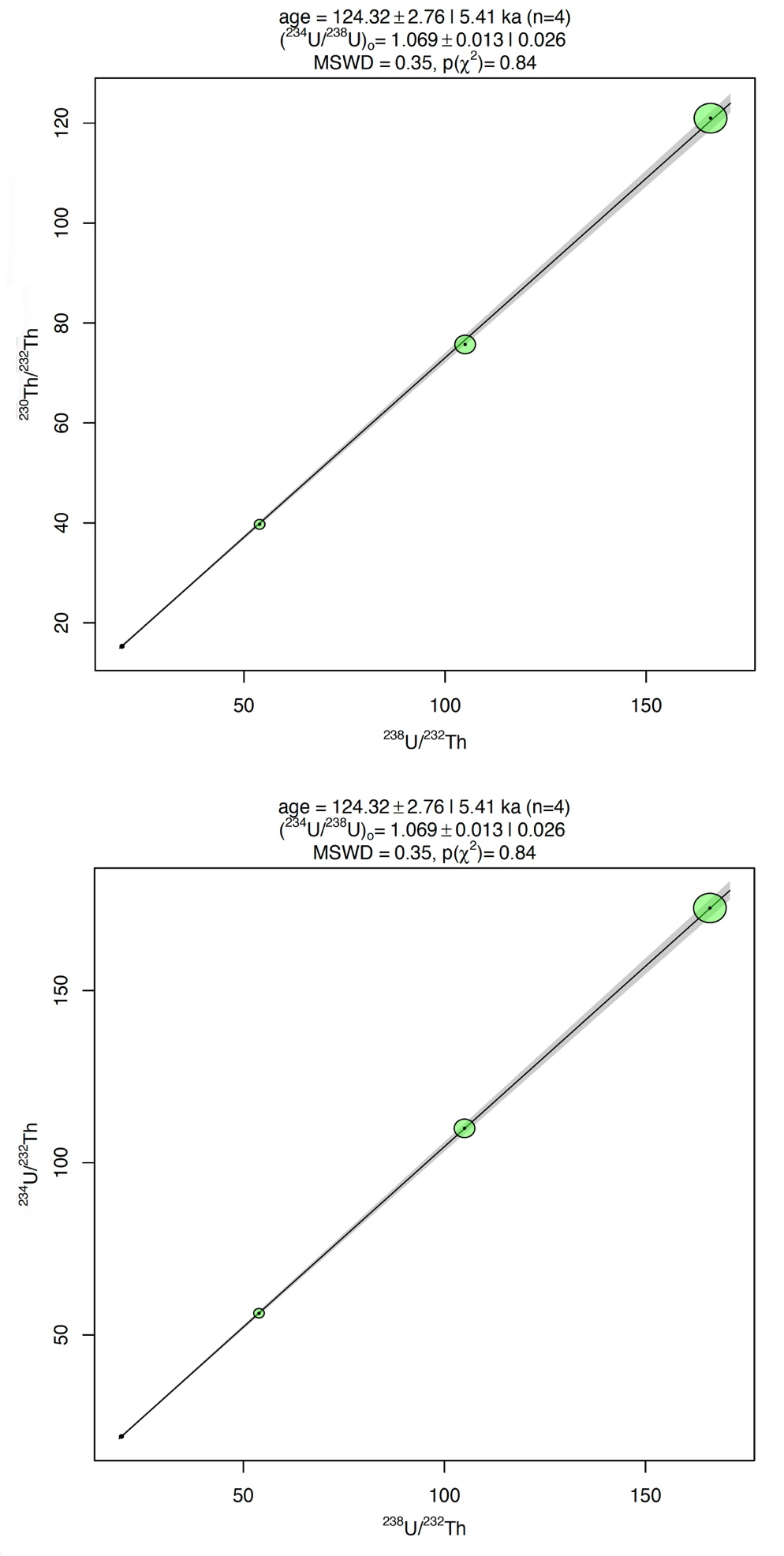

Supplement: Supplementary file 4 — Data S4. ‘Rosholt diagrams’ to the U/Th age of the dated calcite cement. [file SED-69-2099-s004.docx]
