# Supplementary material for: Identification of a Quaternary rock avalanche deposit (Central Apennines, Italy): Significance for recognition of fossil catastrophic mass‐wasting
Source: Sedimentology. 2022 Mar 29;69(5):2099–130. doi: 10.1111/sed.12984 (PMC9541593; doi:10.1111/sed.12984)
Supplement: Supplementary file 5 — Data S5. Table showing background data and references to the interpretation of the deposits discussed in the text. [file SED-69-2099-s005.docx]

**Table S5**. Locations of comparative deposits mentioned and illustrated in text (see Fig. 11, Fig. 12). LGM=Last Glacial Maximum

| **Location, state**  **Position** | **Type of deposit** | **Figure reference**  **Reference** |
| --- | --- | --- |
|  |  |  |
| Marocche di Dro, Italy.  N 45°59'36'' / E 10°56'33'' | Rock avalanche. | Fig. 11A, 11B  Ivy-Ochs et al. (2017) |
|  |  |  |
| Tamins, Switzerland.  N 46°48'51''/ E 9°24'41'' | Rock avalanche. | Fig. 11C, 11D, 11E  Abele (1997) |
|  |  |  |
| Tschirgant, Austria.  N 47°13'48'' / E 10°50'14'' | Rock avalanche. | Fig. 11F  Patzelt and Poscher (1993), Dufresne et al. (2015) |
|  |  |  |
| Hinterriss, Austria.  N 57°31'15'' / E 11°26'27'' | Basal till of LGM. | Fig. 12A  Costantini and Ortner (2013) |
|  |  |  |
| Urschenbach, Austria.  N 47°19'27'' / E 11°32'54'' | Alluvial fan, post-LGM. | Fig. 12B  Sanders and Ostermann (2011) |
|  |  |  |
| Acqua Grossa near Assergi, Italy.  N 42°26'49'' / E 13°29'35'' | Fossil scree slope. | Fig. 12C  Servizio Geologico d'Italia, Foglio #349 Gran Sasso |
|  |  |  |
| Camarda, Italy.  N 42°23'37'' / E 13°29'49'' | Fossil scree slope. | Fig. 12D  Servizio Geologico d'Italia, Foglio #349 Gran Sasso |
|  |  |  |
| Road to Mola de Lord near Sant Llorenç de Moryuns, Pyrenees, Spain.  N 42°07'43'' / E 1°34'26'' | Syntectonic proximal fluvial conglomerate. | Fig. 12E  Riba (1976) |
|  |  |  |
| Riglos, Pyrenees, Spain.  N 42°21'00'' / E 0°43'45'' | Syntectonic proximal fluvial conglomerate. | Fig. 12F  Lloyd et al. (1998) |

**References**

**Abele, G**. (1997) Rockslide movement supported by the mobilization of

groundwater-saturated valley floor sediments. *Z. f. Geomorph*., **41**, 1–20.

**Costantini, D.** and **Ortner, H.** (2013) Klüfte und Deformationsstrukturen in jungpleistozänen Beckensedimenten des Rißtales, Bayern. *GeoAlp*, **10**, 5-26.

**Dufresne, A., Prager, C.** and **Bösmeier, A.** (2015) Insights into rock avalanche emplacement processes from detailed morpho-lithological studies of the Tschirgant deposit (Tyrol, Austria). *Earth Surf. Proc. Landf*., **41**, 587-602.

**Ivy-Ochs, S., Martin, S., Campedel, P., Hippe, K., Alfimov, V., Vockenhuber, C., Andreotti, E., Carugati, G., Pasqual, D., Rigo, M.** and **Viganò, A.** (2017) Geomorphology and age of the Marocche di Dro rock avalanches (Trentino, Italy). *Quat. Sci. Rev*., **169**, 188-205.

**Lloyd, M.J., Nichols, G.J.** and **Friend, P.** (1998) Oligo-Miocene alluvial fan evolution at the southern Pyrenean thrust front, Spain. *J. Sed. Res*., **68**, 869-878.

**Riba, O**. (1976) Syntectonic unconformities of the Alto Cardener, Spanish Pyrenees: A genetic interpretation. *Sed. Geol*., **15**, 213-233.

**Patzelt, G.** and **Poscher, G.** (1993) Der Tschirgant-Bergsturz. Arbeitstagung 1993 Geologische Bundesanstalt: Geologie des Oberinntaler Raumes - Schwerpunkt Blatt 144 Landeck. Exkursion D: Bemerkenswerte Geologische und Quartärgeologische Punkte im Oberinntal und aus dem äußerem Ötztal. Geologische Bundesanstalt, Vienna, 206–213.

**Sanders, D.** and **Ostermann, M.** (2011) Post-last glacial alluvial fan and talus slope associations (Northern Calcareous Alps, Austria): A proxy for Late Pleistocene to Holocene climate change. *Geomorphology*, **131**, 85-97.
